# Cross-Validation of Generic Risk Assessment Tools for Animal Disease Incursion Based on a Case Study for African Swine Fever

**DOI:** 10.3389/fvets.2020.00056

**Published:** 2020-02-18

**Authors:** Clazien J. de Vos, Rachel A. Taylor, Robin R. L. Simons, Helen Roberts, Cecilia Hultén, Aline A. de Koeijer, Tapani Lyytikäinen, Sebastian Napp, Anette Boklund, Ronald Petie, Kaisa Sörén, Manon Swanenburg, Arianna Comin, Leena Seppä-Lassila, Maria Cabral, Emma L. Snary

**Affiliations:** ^1^Department of Bacteriology and Epidemiology, Wageningen Bioveterinary Research (WBVR), Wageningen University & Research, Lelystad, Netherlands; ^2^Department of Epidemiological Sciences, Animal and Plant Health Agency (APHA), Weybridge, United Kingdom; ^3^Department for Environment, Food & Rural Affairs (Defra), London, United Kingdom; ^4^National Veterinary Institute (SVA), Uppsala, Sweden; ^5^Finnish Food Authority (Ruokavirasto), Helsinki, Finland; ^6^Centre de Recerca en Sanitat Animal (CReSA IRTA-UAB), Bellaterra, Spain; ^7^Department of Veterinary and Animal Sciences, Section for Animal Welfare and Disease Control, University of Copenhagen, Frederiksberg, Denmark

**Keywords:** African swine fever, cross-validation, livestock diseases, generic model, introduction risk, model uncertainty, risk assessment

## Abstract

In recent years, several generic risk assessment (RA) tools have been developed that can be applied to assess the incursion risk of multiple infectious animal diseases allowing for a rapid response to a variety of newly emerging or re-emerging diseases. Although these tools were originally developed for different purposes, they can be used to answer similar or even identical risk questions. To explore the opportunities for cross-validation, seven generic RA tools were used to assess the incursion risk of African swine fever (ASF) to the Netherlands and Finland for the 2017 situation and for two hypothetical scenarios in which ASF cases were reported in wild boar and/or domestic pigs in Germany. The generic tools ranged from qualitative risk assessment tools to stochastic spatial risk models but were all parameterized using the same global databases for disease occurrence and trade in live animals and animal products. A comparison of absolute results was not possible, because output parameters represented different endpoints, varied from qualitative probability levels to quantitative numbers, and were expressed in different units. Therefore, relative risks across countries and scenarios were calculated for each tool, for the three pathways most in common (trade in live animals, trade in animal products, and wild boar movements) and compared. For the 2017 situation, all tools evaluated the risk to the Netherlands to be higher than Finland for the live animal trade pathway, the risk to Finland the same or higher as the Netherlands for the wild boar pathway, while the tools were inconclusive on the animal products pathway. All tools agreed that the hypothetical presence of ASF in Germany increased the risk to the Netherlands, but not to Finland. The ultimate aim of generic RA tools is to provide risk-based evidence to support risk managers in making informed decisions to mitigate the incursion risk of infectious animal diseases. The case study illustrated that conclusions on the ASF risk were similar across the generic RA tools, despite differences observed in calculated risks. Hence, it was concluded that the cross-validation contributed to the credibility of their results.

## Introduction

Increasing globalization and international trade contribute to rapid expansion of infectious animal diseases, as illustrated by the recent outbreaks of bluetongue (BT), African swine fever (ASF), lumpy skin disease (LSD), and peste des petits ruminants (PPR) in Europe (1–3). Introduction of exotic animal diseases into naive livestock populations can result in large-scale epidemics with serious economic and socio-ethical impact (4–6). Hence, preparedness is warranted to prevent, detect, and control outbreaks of exotic animal diseases. To make decisions on risk management of exotic animal disease threats, it is necessary to know which animal diseases pose the highest threats and should therefore deserve more attention.

Risk assessment is a useful tool for prioritization of diseases with respect to their incursion risk, the results of which can be used to assign resources for prevention and surveillance to those diseases posing the highest risk or to identify targets for additional research. Most commonly, risk assessments are developed to assess the risk for a single disease and introduction pathway. In recent years, however, several generic risk models or frameworks have been developed that can be applied to assess the incursion risk for multiple animal diseases (7–15). In contrast to bespoke models, these generic risk assessment (RA) tools allow for a more rapid response to a variety of newly emerging or re-emerging diseases.

Generic RA tools, however, tend to have a lower resolution in their algorithms to allow the assessment of risk over multiple diseases that differ with respect to the species of animals affected, transmission modes, and epidemiological and economic impact. Furthermore, uncertainty and variability are not always considered in much detail. To parameterize generic RA tools, global databases are preferred, as these contain information over multiple countries, diseases, and/or introduction pathways. Application of results from generic RA tools vary from a rapid response to new emergencies to horizon scanning and prioritization of diseases, pathways, or regions.

A shared problem of generic tools with bespoke models is the validation of their results. Most import risk assessments estimate the probability of rare events occurring in an ever-changing world, leaving the use of a long range of historical data for this purpose useless. Validation of import risk assessments is, nevertheless, an important step in their development to ensure the plausibility of results. Sensitivity analysis to address parameter uncertainty by varying the values of uncertain input parameters in a plausible range contributes to the internal validation of risk assessments (16, 17). With several generic RA tools having been developed in recent years in Europe that all can be used to assess the incursion risk of notifiable animal diseases such as ASF, LSD, and BT, opportunities arise to address model uncertainty by comparing results obtained with different tools. The objective of this study was to explore the opportunities for cross-validation of generic RA tools, where cross-validation was defined as the validation of model results by comparing them with results of other models that addressed the same question. ASF was selected as a case study given its rapid spread in Europe in recent years (18–20).

ASF is a viral disease of pigs and wild boar caused by the ASF virus, the only member of the family *Asfarviridae* (21–23). ASF virus (genotype 2) was introduced into the Caucasus region in 2007 and has subsequently spread into Belarus, Russia and Ukraine and then to the European Union in 2014 with infection having been reported from Estonia, Latvia, Lithuania, Poland, Czech Republic, Romania, Hungary, Bulgaria, and Belgium in recent years (2, 3). Prevention of ASF introduction is a high priority for European countries still free from the disease as introduction of the disease can have severe consequences for the domestic pig sector and the wild boar population due to an extremely high case fatality rate (up to 100%) (24). Rigorous measures are needed to control the disease including culling of infected herds and movement bans, and there are difficulties in eradicating the disease once it has established itself in the wild boar population (24, 25). There is currently no vaccine available against ASF infection and infection continues to spread through various pathways, including movement of infected wild boar and human mediated routes.

Results of risk assessment studies are an important input into risk management decisions to prevent ASF spread to new regions and several bespoke models for ASF have been developed in the last decade (26–31). All generic RA tools of this study can also be used to assess the ASF incursion risk with the major advantage that results can easily be updated when outbreaks are reported from new regions in Europe. However, because of their generic character, validation is an even more essential step to ensure plausibility of results.

To cross-validate the generic RA tools of this study, all tools were used to assess the incursion risk of ASF to two European countries, the Netherlands and Finland. These countries were chosen because of their opposite risk profiles when considering their ASF risk, with the Netherlands being a trading country not only exporting millions of pigs annually, but also importing over a million of live pigs each year (32), whereas Finland has hardly any international trade in live pigs (33). Finland, on the contrary, was geographically much closer to observed outbreaks of ASF in Europe at the time this study was initiated (before the ASF outbreaks in wild boar in Belgium in September 2018).

## Materials and Methods

### Definitions

Although an assessment of the incursion risk would ideally address both the probability of incursion of disease and its subsequent consequences, not all generic RA tools of this study had incorporated a consequence assessment. Therefore, in this study *incursion risk* is used as a generic term to indicate any metric to estimate the risk of exotic diseases entering a new territory, varying from entry only to a full risk assessment including epidemiological and economic consequences.

Regarding the probability of incursion, four separate steps are distinguished in this study. In the *entry* step the probability that an infectious agent enters a new territory (hereafter called: target area) by any pathway is evaluated, without assessing subsequent exposure of susceptible animals in the target area via this pathway. In the *exposure* step the probability that a susceptible native host animal in the target area is exposed to the infectious agent is evaluated given its entry into the target area, without assessing the probability that such exposure would result in infection. In the first *infection* step the probability that contact with the infectious agent results in infection of a first native host in the target area (= index case) is evaluated given its entry into the target area and exposure of the host animal. In the *establishment* step the probability that the infectious agent will start spreading in the target area is evaluated given it has entered the target area and resulted in a first infection of native host animals. Establishment is an important step when evaluating the incursion risk of vector-borne diseases, where an infection of a native host animal can be a dead-end if, e.g., no competent vectors are present in the target area or if climatic conditions impede subsequent transmission.

Consequences of disease incursion have been separated into epidemiological and economic consequences. *Epidemiological consequences* have been defined as the expected spread of the infectious agent in the native (susceptible) population in the target area or further geographical spread from the target area to new regions, considering, e.g., the epidemic size and geographic area affected. *Economic consequences* have been defined as the expected monetary losses resulting from an outbreak with the infectious agent in the target area due to, e.g., morbidity and mortality, production losses, control measures and trade restrictions.

### Generic Risk Assessment Tools

Seven generic RA tools, all developed by the G-RAID^1^ consortium (34), were included in the cross-validation study. Selection criteria for inclusion were (i) the tool was developed to assess the incursion risk of multiple diseases rather than a single disease, and (ii) the tool focused on the veterinary risk of animal diseases rather than public health. Although all seven tools can be used to address the incursion risk of exotic animal diseases, they were originally developed for different purposes ranging from immediate response to new disease events to prioritization of diseases and horizon scanning. As a consequence, input, algorithms, and endpoints of the tools differed. The seven generic RA tools included two quantitative tools (SPARE, COMPARE), four semi-quantitative tools (RRAT, MINTRISK, IDM, NORA) and one qualitative tool (SVARRA). A brief overview of all tools is given in Table 1. More details are available in Supplementary Material 1.

**Table 1:** Brief outline of seven generic RA tools.

**RA tool**	**Objective**	**Prioritization**	**Data available in tool**	**Number of diseases in tool**	**Number of pathways in tool**	**Variability/uncertainty included**	**Software**	**Developer**	**More information**
SPARE	Early warning of disease incursion risks	Target areas	Yes	4	5	None	R	APHA, UK	(14, 35)
COMPARE	Identification of hotspots for risk-based surveillance	Target areas, pathways	No	3	5	Variability	R	APHA, UK	(15, 36–38)
RRAT	Identification of high priority exotic notifiable diseases	Pathways, diseases	Yes	10	3	None	R, SQLite	WBVR, NL	(39)
MINTRISK	Comparison and prioritization of vector-borne diseases	Target areas, diseases	No	NA^a^	NA^b^	Uncertainty	C#, Visual Studio	WBVR, NL	(10, 11, 40, 41)
IDM	Identification of high priority exotic notifiable diseases	Diseases	Yes	34	7	None	Excel	Defra, UK	(8, 11)
NORA	Rapid risk assessment to respond to new disease events	Pathways	No	NA^a^	9	None	Excel	Ruokavirasto, FI	(12)
SVARRA	Rapid risk assessment to respond to new disease events	Pathways	No	NA^a^	8	Uncertainty	Word, Excel	SVA, SE	(11)

a *These tools have no underlying database with disease-specific data and can evaluate any disease*.

b *MINTRISK can evaluate any pathway*.

All seven RA tools were built to be flexible with respect to the animal diseases to be evaluated, although MINTRISK was primarily designed to assess the risk of vector-borne diseases. The total number of diseases evaluated so far with each of the tools varies greatly, as does the level of resources (expertise, data, time) needed to complete a risk assessment. For all tools, the assessment is less rapid if the disease has not been evaluated before with the tool, because additional data collection and parameterization is required. The RRAT and the IDM tool have the data required to perform the risk assessment readily available in the tool for a multitude of diseases. SPARE and COMPARE have only been parameterized for a few diseases, although the data available on disease prevalence worldwide in these tools can theoretically be used to assess the risk of any OIE-listed disease. MINTRISK, NORA, and SVARRA come mostly without underlying databases and have to be filled by the risk assessor.

The tools also differ widely with respect to the number of introduction pathways that can be evaluated (Table 2). Legal trade in live animals and imports of products of animal origin are considered by each tool, although these pathways were not always consistently defined across the tools. For example, some tools consider trade in livestock animals only, whereas other tools also consider trade in pets and exotic animals. Most tools also address windborne vector spread and wild animal dispersion including migratory birds. All tools but MINTRISK have predefined pathways built in. MINTRISK asks the risk assessor to define relevant pathways for the disease considered, either related to vertebrate host animals and their products, vectors, or humans. In general, the tools with relatively uncomplicated algorithms, such as the qualitative tool SVARRA and the semi-quantitative tools IDM and MINTRISK, are most flexible to include additional pathways.

**Table 2:** Introduction pathways embedded in each of the generic RA tools.

**PATHWAY**	**SPARE**	**COMPARE**	**RRAT**	**MINTRISK^a^**	**IDM**	**NORA**	**SVARRA**
Live animals	X	X	X	X	X	X	X
Products of animal origin	X	X	X		X	X	X
Germplasm			X		X	X	X
Vectors	X	X			X	X	X
Wildlife	X	X		X	X	X	X
Human travel	X	X				X	X
Transport					X	X	X
Laboratory material and samples					X		
Feed and bedding						X	X
Airborne spread						X	

a *MINTRISK can deal with any pathway, but none are embedded in the tool; these are the pathways that were evaluated for the ASF case study*.

**Figure 1:**
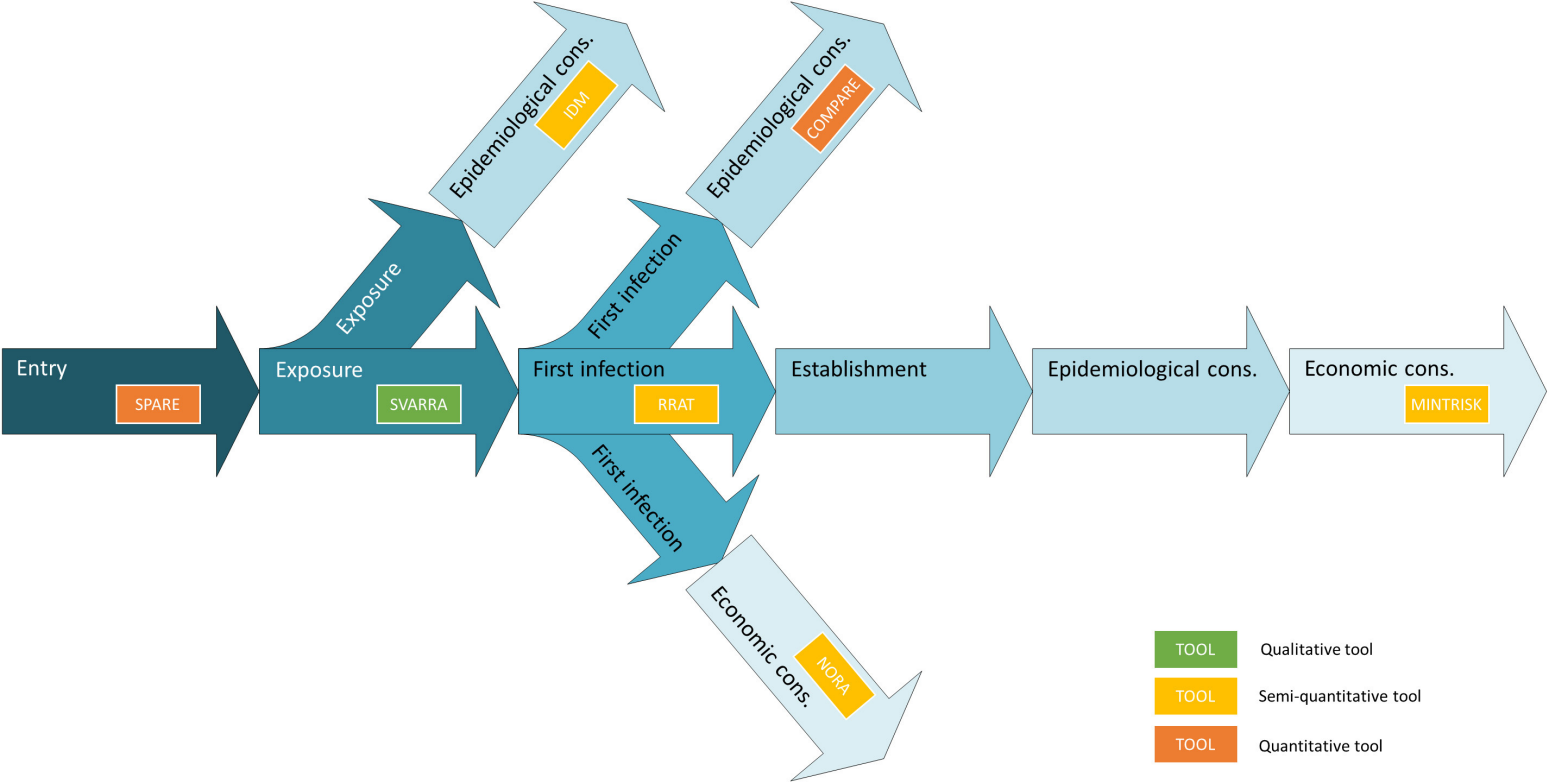
Outline of the steps involved in assessing disease incursion risks indicating the entirety of the generic RA tools.

The outline of all seven generic RA tools is primarily based on the OIE import risk assessment framework (42). However, the RA tools differ widely with respect to the steps that are included to assess the disease incursion risk (Figure 1). Endpoints of the tools differ alike. MINTRISK is the most complete tool considering entry up to establishment, and epidemiological and economic consequences. SPARE on the contrary only considers entry. All tools use the basic principles of the Binomial model (43) to assess entry of pathogens into new areas, combining information on pathway numbers (*N*) with probabilities of infection (*p*) based on prevalence levels. All tools that evaluate epidemiological consequences (COMPARE, MINTRISK, IDM) do so by estimating the basic reproductive number *R*_0_ (44) and implementing it into their model calculations.

Only a few of the generic RA tools have embedded uncertainty and/or variability in their risk assessment. COMPARE is the only tool addressing variability in calculating the incursion risk using stochastic calculations, including primarily the variability in disease prevalence in traded animals/products but also variability in other disease parameters. MINTRISK and SVARRA are the only tools that explicitly ask the risk assessor for his/her uncertainty in estimating the input parameter values, and only MINTRISK uses stochastic simulation to address this uncertainty. NORA also acknowledges that risk assessors cannot be expected to know everything and offers the “I don't know” option in answering the questions. This uncertainty is reported in the results of the tool by counting the number of questions that were given this answer. Despite the fact that uncertainty is not embedded in the other RA tools, most of them offer the opportunity to consider uncertainty via scenario analysis.

### Risk Question and Scenarios

**Figure 2:**
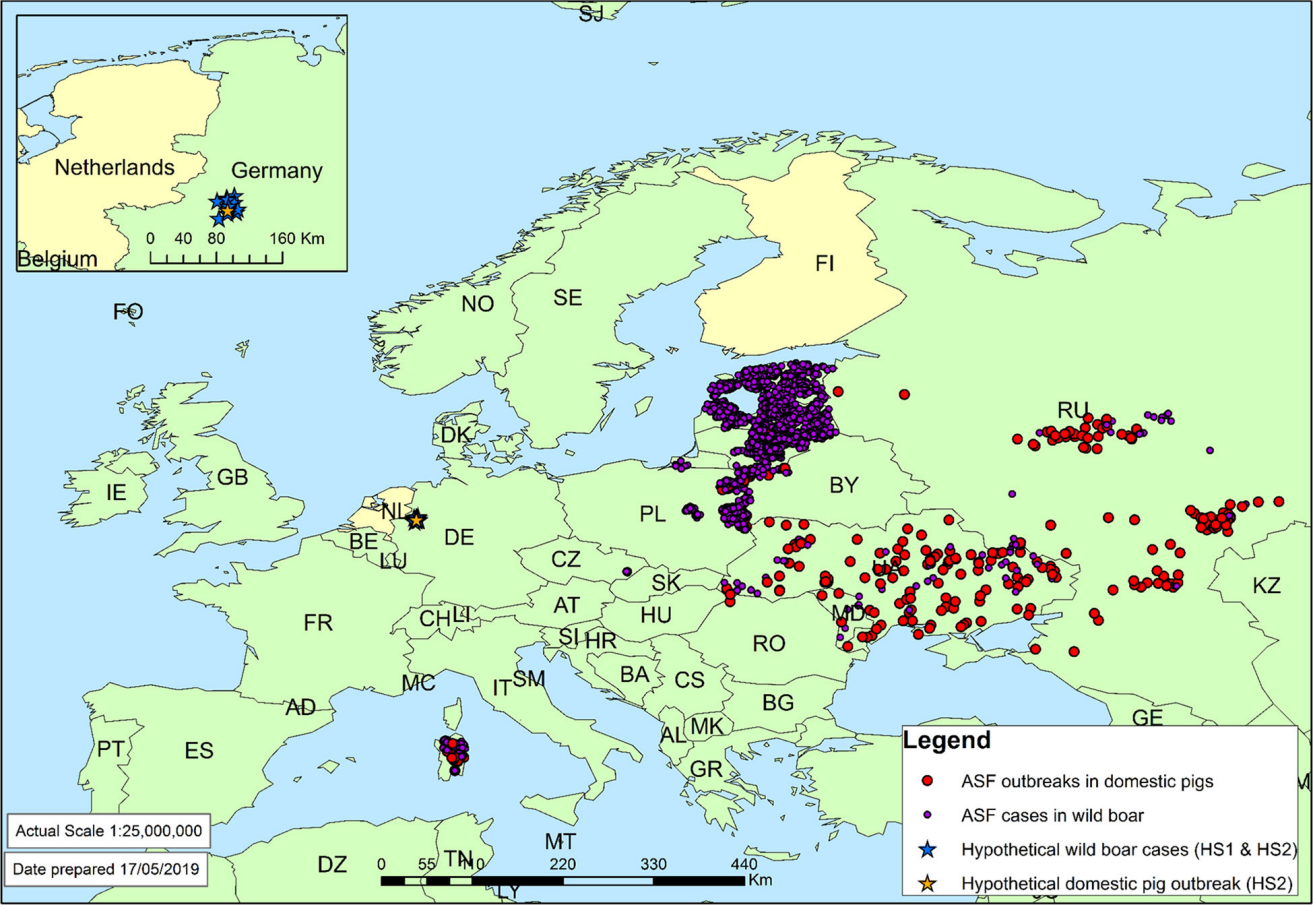
ASF cases in domestic pigs and wild boar in 2017 as reported to the OIE (2). Target countries of the case study (the Netherlands and Finland) have been colored yellow. Inset: location of hypothetical ASF cases in Germany.

To explore the opportunities for cross-validation of the generic RA tools, all seven tools were used to assess the risk for a selected case study considering several African swine fever scenarios. The risk question considered the ASF virus strain responsible for European cases in 2017 as the hazard and was formulated as: Given the history of ASF cases reported in Europe in 2017, as well as trade patterns in 2017, what is the predicted incursion risk of ASF to (a) the Netherlands and (b) Finland from any country where the virus strain circulates? In addition, the same question was answered considering two hypothetical scenarios in which ASF cases were reported in Germany. In the first hypothetical scenario (HS1), it was assumed that on 30/12/2017 10 separate cases of wild boar found dead, infected with ASF, were reported from the Munster region of Germany at a distance of ~50 km from the Dutch border (Figure 2). In the second hypothetical scenario (HS2), it was assumed that on 30/12/2017, as per scenario HS1, ten separate cases of ASF in wild boar were reported from the Munster region of Germany, and that one outbreak on a single commercial mixed (breeding and fattening) farm had been reported in the same region with 2,500 pigs on it, 18 of which were found infected and all 2,500 were immediately culled (Figure 2). In both hypothetical scenarios, the history of ASF outbreaks and reported trade patterns were assumed to be the same as in the 2017 scenario. Furthermore, hypothetical ASF cases in scenarios HS1 and HS2 were assumed to behave in a similar way to the other cases in Europe in 2017 with regards to characteristics such as infectious period and transmission rate.

### Input Data for the African Swine Fever Case Study

The majority of the data required for the generic RA tools can be broken down into four categories: (1) pathway movements from source areas to the target area; (2) disease prevalence in source areas; (3) susceptible animals in the target area; and (4) disease-specific parameters. For the case study, input data used by the tools were harmonized wherever possible to ensure that differences in results would result from model uncertainty rather than input parameter uncertainty. An overview of input data shared between the tools is available in Supplementary Material 2.

#### Pathway Movements

Data on pathway movements are relevant to the entry stage of the risk assessment and focus on how many units (animals, products etc.) will reach the target area from different source areas, regardless of whether or not they are infected. All generic RA tools derive data on pathway movements predominantly from global databases on international trade. For the legal trade pathways, all of the tools can use one or more of TRACES (45), Comext (33), or Comtrade (46). For movement of wild animals and illegal trade, global datasets are obviously not possible to obtain. However, most tools incorporate global datasets of travel statistics (47) or population abundance maps in order to either model or estimate a score for how much illegal trade or wild animal movements are to be expected.

For the case study, all of the generic RA tools used Comext trade data for 2017 (33) to assess the numbers of animals, products (including germplasm and laboratory material), and travelers entering the Netherlands and Finland. However, the selection of CN codes (Combined Nomenclature) (48, 49) included in the tools depended on how pathways were defined and was not the same across all tools, especially when considering the legal trade in animal products. Population abundance maps (50) were used to either model or estimate a score for how many wild boar movements were to be expected. The quantitative tools (SPARE, COMPARE) applied mechanistic approaches (in which a complex problem is broken down to the underlying mechanisms) to determine the movement of wild boars and harmonized the parameters within these tools as far as possible, e.g., percentage of wild boars that move long distances and how far they move.

#### Disease Prevalence

Data on disease prevalence are also relevant to the entry stage of the risk assessment and are used to estimate the probability that animals/products are infected. Disease prevalence in source areas is primarily based on information on disease occurrence derived from the World Animal Health Information System (WAHIS) (2), although Empres-i (51) and the Animal Disease Notification System (ADNS) (52) are also used across the tools. Furthermore, the more qualitative tools might also include information derived from reports and mailings from the European Commission.

For the case study, all generic RA tools were fed with data from WAHIS (2) on the number of ASF outbreaks and the number of cases by species (domestic pigs, wild boar) on the country level for 2017 and previous years. An overview of ASF cases reported to OIE in 2017 is given in Figure 2. To estimate disease prevalence in the source countries, information on pig and wild boar populations in those countries were also derived from WAHIS. The RA tools used, however, different approaches to convert the WAHIS data into disease prevalence estimates. Whereas some of them included all historical data available from the OIE website, others only considered information on disease outbreaks in a recent period (e.g., a 1-year period or the high-risk period, i.e., the period from introduction of disease into an area until its detection). Furthermore, some tools had built-in algorithms to correct for non-reporting or underreporting, or to include a probability of disease presence despite current absence, whereas other tools based their prevalence estimates on the actual situation reported to OIE only. In addition, the more qualitative tools had the ability to include additional information derived from, e.g., ADNS and reports and mailings from the European Commission, especially when assessing the infection probabilities of wild boar populations.

#### Susceptible Animals

Data on susceptible animals relate to the probabilities of disease transmission to susceptible populations in the target area. For this category of data input, more variation is observed between the seven tools due to contrasting choices made to model contact between susceptible animals and infected animals/products. Whereas some tools include detailed data on number of animals and farms or livestock densities in the target area derived from global databases such as WAHIS (2) and FAOSTAT (53), other tools distinguish between different farm types based on size and biosecurity level or include information on disease susceptibility only. One of the tools, SPARE, has not included information on susceptible animals in the target area at all, because it only evaluates entry of the pathogen, and not subsequent exposure or infection.

As a result, harmonization of data on susceptible animals in the Netherlands and Finland was difficult. COMPARE used maps on wild boar abundance (50), whereas most tools did not need data at this spatial scale and used data on wild boar presence and abundance from Dutch and Finnish sources (54–56).

#### Disease-Specific Parameters

The category of disease-specific input parameters includes all parameters specific to the disease, such as duration of the latent and infectious period, transmission probabilities, severity of clinical signs, test sensitivities, and decay rates in products. For this category of data input, the tools differ in whether and how they incorporate these parameters; however, all of them primarily used published literature and expert opinion to find relevant parameter values.

For the case study, disease-specific parameters were sourced individually by each tool from published literature [e.g., (57–61)] and expert opinion. Parameter values were shared to enable harmonization of input data over the RA tools if the same input parameters were used (Supplementary Material 2).

### Comparison of Results

Although ideally both the probability of incursion of disease and its consequences are evaluated in assessing the incursion risk of exotic diseases, results of the generic RA tools for the ASF case study could only be compared for the probability of incursion, because consequences were only assessed by some tools. A comparison of absolute results obtained by the seven generic RA tools was not possible though, because endpoints for the probability of incursion varied from entry to establishment (Table 3). In addition, the tools had different output parameters (Table 3) and evaluated different numbers and types of introduction pathways (Table 2). All tools assessed the ASF risk from legal trade in live animals and six out of seven tools assessed trade in animal products (all but MINTRISK) and wild boar movements (all but RRAT). Therefore, for each tool, relative risks were calculated by country and scenario for the three pathways most in common to enable comparison of results. These were compared to see if the tools agreed on the directions and magnitudes of the relative risks resulting in a similar prioritization of countries and scenarios. In addition the pathways within each RA tool were compared against each other to identify the pathways contributing most to the ASF risk to the Netherlands and Finland.

**Table 3:** Risk of ASF incursion as evaluated by the seven generic RA tools.

**RA tool**	**Endpoint**	**Output type**	**Output parameter**
SPARE	Entry	Quantitative	Number of entries per year
COMPARE	First infection	Quantitative	Annual probability
RRAT	First infection	Semi-quantitative	Risk score (from 0 to 1)
MINTRISK	Establishment	Semi-quantitative	Annual rate
IDM	Exposure	Semi-quantitative	Risk score (from 0 to 60)
NORA	First infection	Semi-quantitative	Risk score (from 0 to 1)
SVARRA	Exposure	Qualitative	Qualitative probability level

The relative risk across the two countries (*RR*_*c*_*ij*__) for each tool *i* was calculated for each pathway *j* as:

$$\begin{array}{l} RR_{c_{ij}}=R_{NLD_{ij}}/R_{FIN_{ij}} \end{array}$$

where *R*_*NLD*_*ij*__ is the calculated risk by tool *i* to the Netherlands for pathway *j* and *R*_*FIN*_*ij*__ the calculated risk by tool *i* to Finland for pathway *j*. Calculations were done for the baseline scenario (2017 situation) only.

Relative risks across scenarios (*RR*_*HS1*_*ijk*__ and *RR*_*HS2*_*ijk*__) for each tool *i* were calculated for each pathway *j* and each country *k* as:

$$\begin{array}{l} RR_{{HS1}_{ijk}}=R_{{HS1}_{ijk}}/R_{Base_{ijk}}\text{ and }RR_{{HS2}_{ijk}}=\;R_{{HS2}_{ijk}}/R_{Base_{ijk}} \end{array}$$

where *R*_*Base*_*ijk*__ is the calculated risk by tool *i* for pathway *j* and country *k* for the baseline scenario (2017 situation), *R*_*HS*1_*ijk*__ is the calculated risk by tool *i* for pathway *j* and country *k* for the first hypothetical scenario (ASF in wild boar in Germany), and *R*_*HS*2_*ijk*__ is the calculated risk by tool *i* for pathway *j* and country *k* for the second hypothetical scenario (ASF in wild boar and domestic pigs in Germany).

In order to calculate relative risks for the qualitative RA tool SVARRA, qualitative probability levels were converted to numerical values using a log-scale, where negligible = 1, very low = 10, low = 100, etc. The in-between probability level negligible/very low was given a numerical value of $\sqrt{10}$. All other RA tools provided numerical results, either representing absolute risk estimates or semi-quantitative risk scores (see Table 3), making it possible to calculate relative risks. The only exceptions were scenarios resulting in a negligible or zero result. To enable the calculation of relative risks, negligible and zero results were set equal to 10^−10^, based on the lowest results that were calculated by the tools (7 × 10^−10^ for the risk of the live animal trade pathway to Finland as calculated by RRAT).

## Results

### Relative Risks Across Countries

**Figure 3:**
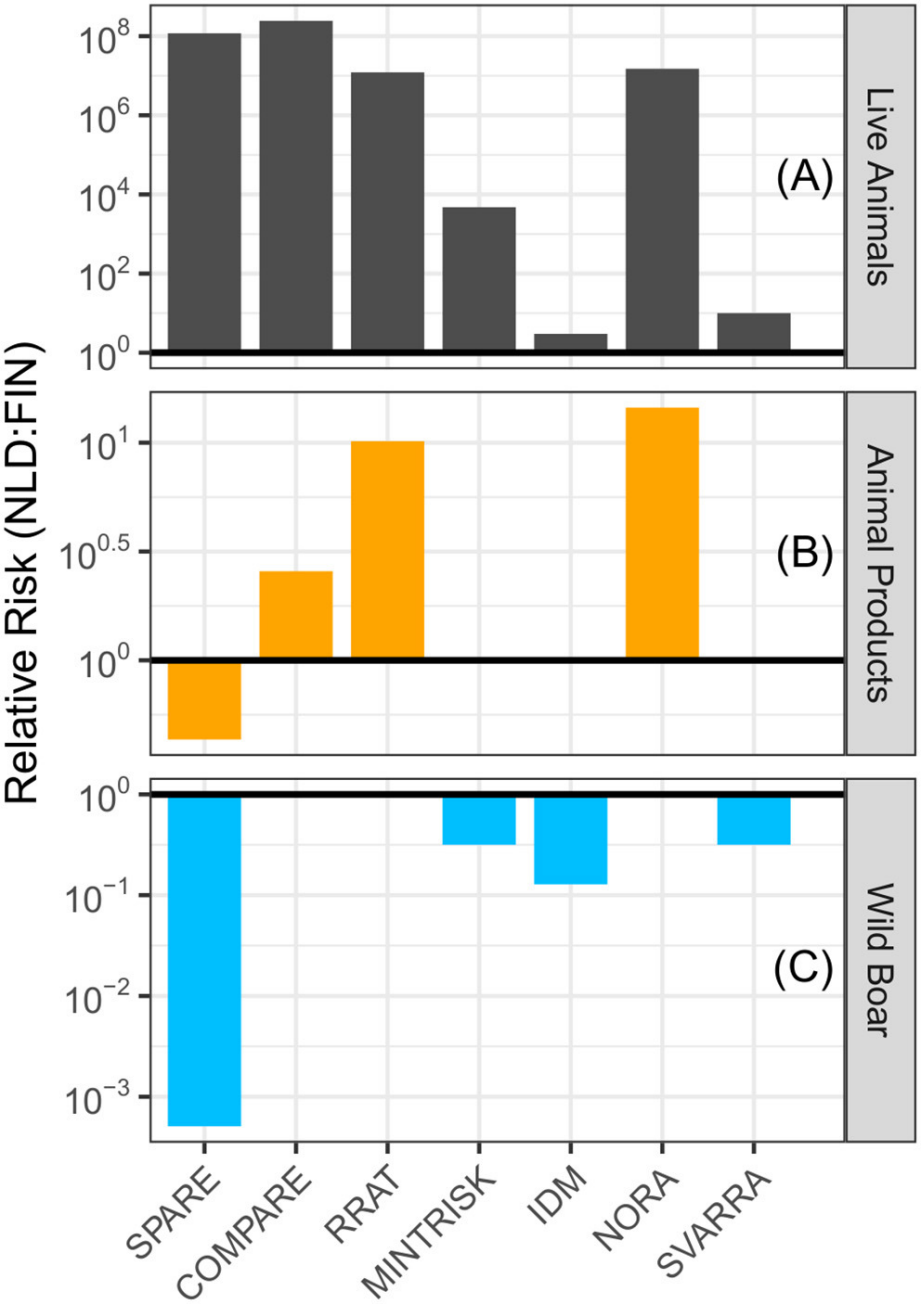
Relative risk of introducing ASF into the Netherlands compared to Finland in the baseline scenario (2017 situation) by **(A)** trade in live animals, **(B)** trade in animal products, and **(C)** movement of wild boar. A relative risk above 1 (bold line) denotes the Netherlands has a higher risk than Finland, while a relative risk below 1 denotes Finland has a higher risk. Please note the different scales used on the y-axes.

For each of the generic RA tools, the ASF incursion risk to the Netherlands was compared to Finland for the pathways (a) trade in live animals, (b) trade in animal products, and (c) wild boar movements. Results for the baseline scenario (2017 situation) are given in Figure 3. A calculated relative risk above 1 indicates that the ASF risk was higher to the Netherlands than Finland. From Figure 3A it can be seen that, for each of the RA tools, the evaluated risk of the live animal trade pathway was higher to the Netherlands than Finland. In particular SPARE, COMPARE, RRAT and NORA predicted a much higher risk (i.e., over 10^7^ times higher).

Six of the seven RA tools evaluated the animal products pathway (MINTRISK did not) (Figure 3B). COMPARE, RRAT, and NORA predicted a higher incursion risk to the Netherlands than Finland for this pathway, although differences in risks were much smaller than for the live animal trade pathway. IDM and SVARRA predicted an equivalent risk for both countries, whereas SPARE uniquely predicted that the risk was lower to the Netherlands than Finland.

Again, six of the seven RA tools evaluated the wild boar pathway (RRAT did not) (Figure 3C). COMPARE and NORA predicted an equivalent, very low to negligible incursion risk resulting from this pathway to both countries. All other tools had a relative risk below 1, which indicates that the predicted ASF risk was higher to Finland than the Netherlands.

### Relative Risks Across Scenarios

**Figure 4:**
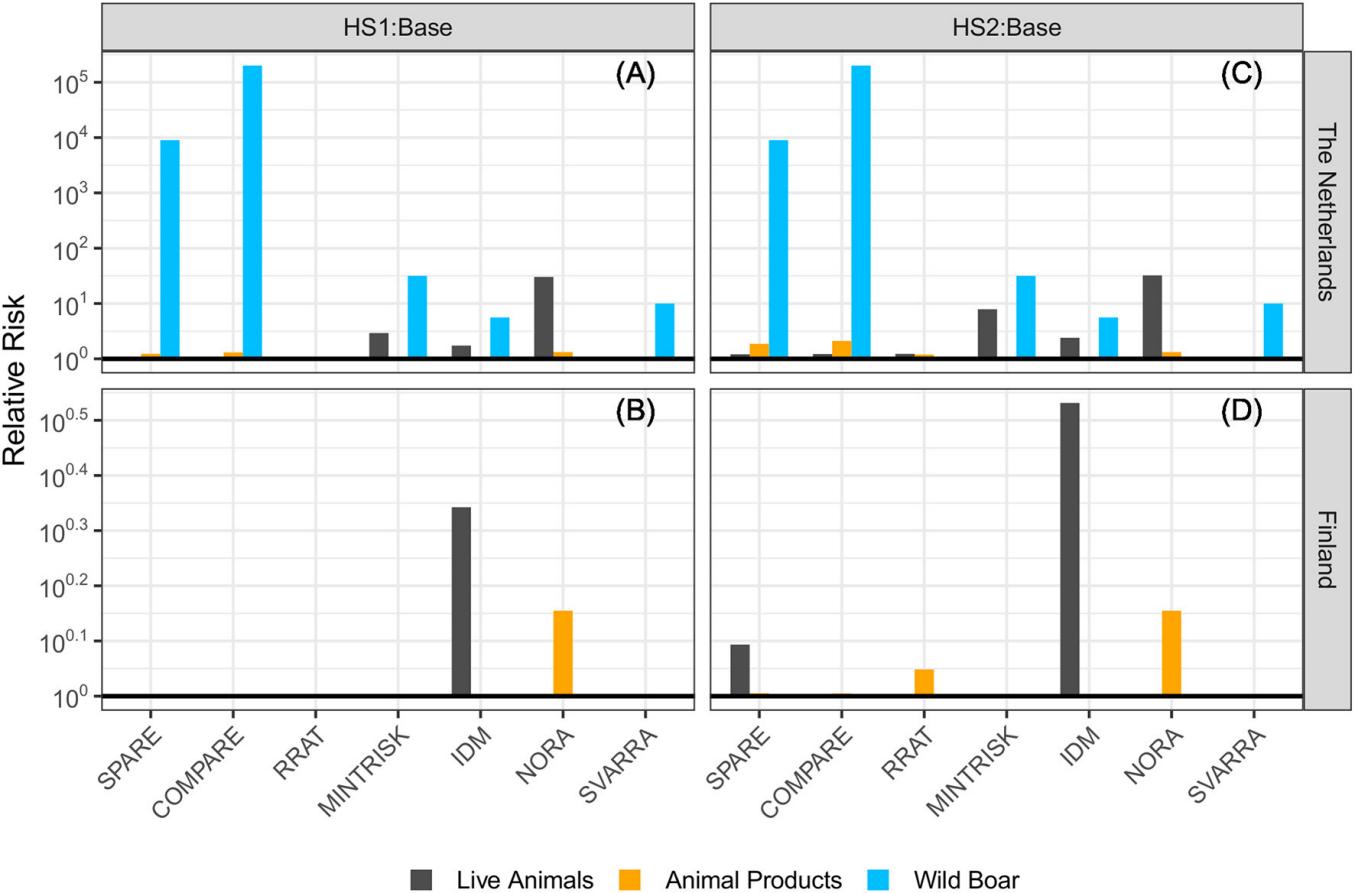
Relative risk of introducing ASF into **(A)** the Netherlands and **(B)** Finland in the hypothetical scenario with ASF reported in wild boar in Germany (HS1) and **(C)** the Netherlands and **(D)** Finland with ASF reported in wild boar and domestic pigs in Germany (HS2) compared to the baseline scenario (2017 situation). A relative risk of 1 (bold line) denotes no differences in risks among the scenarios. Please note the different scales used on the y-axes.

For each of the generic RA tools, the ASF incursion risk of both hypothetical scenarios was compared to the baseline scenario. Results for the Netherlands and Finland are given in Figure 4. A calculated relative risk above 1 indicates that the ASF risk was higher in the hypothetical scenario than in the baseline scenario.

The hypothetical situation in which ASF cases were reported in wild boar in Germany (HS1) resulted in an increased risk to the Netherlands compared to the baseline scenario, especially for the wild boar pathway. In particular SPARE and COMPARE predicted a much higher risk (i.e., over 10^3^ times higher). NORA was the only tool of those evaluating the wild boar pathway that did not predict an increased risk for this pathway in HS1 to the Netherlands. The addition of a single ASF outbreak at a domestic pig farm (HS2) resulted in a slightly increased risk to the Netherlands for the live animal trade pathway compared to HS1 for MINTRISK, IDM and NORA, and a slightly increased risk for both the live animal trade pathway and the animal products pathway for SPARE, COMPARE and RRAT. SVARRA indicated that the ASF outbreak at a domestic farm in Germany (HS2) did not further increase the ASF risk to the Netherlands compared to HS1.

Most RA tools agreed that the presence of ASF in wild boar in Germany (HS1) did not increase the risk to Finland compared to the baseline scenario. Only IDM and NORA predicted a slightly increased risk for the live animal trade pathway and the animal products pathway, respectively. The addition of a single ASF outbreak at a domestic pig farm (HS2) resulted in a slightly increased risk to Finland for the live animal trade pathway compared to HS1 for SPARE and IDM, and a slightly increased risk for the animal products pathway for SPARE, COMPARE, and RRAT. MINTRISK and SVARRA indicated that the presence of ASF in wild boar and/or domestic pigs in Germany did not increase the ASF risk to Finland compared to the baseline scenario.

### Pathway Contribution

Table 4 presents an overview of the three most commonly investigated pathways in the tools (i.e., trade in live animals, trade in animal products and wild boar movements), indicating the pathway that contributed most to the ASF incursion risk to the Netherlands and Finland in the baseline scenario and the two hypothetical scenarios. Results for SPARE and IDM are not included here, because a relative comparison of estimated pathway risks was not possible for these tools. In SPARE the units in which the risks are expressed differ over the pathways being numbers of infected animals for the live animal trade and wild boar pathway and numbers of infected kilograms for the animal products pathway (Supplementary Material 3). In IDM the semi-quantitative risk scores assigned to each of the pathways have not been scaled to allow for a comparison between the pathways.

**Table 4:** Pathways contributing most to the ASF incursion risk for each tool^a^ per country and scenario (indicated in red)^b^.

**RA TOOL**	**Scenario**	**The Netherlands**			**Finland**		
		**Live animals**	**Animal products**	**Wild boar**	**Live animals**	**Animal products**	**Wild boar**
COMPARE	Baseline						
	HS1						
	HS2						
RRAT	Baseline						
	HS1						
	HS2						
MINTRISK	Baseline						
	HS1						
	HS2						
NORA^c^	Baseline						
	HS1						
	HS2						
SVARRA^d^	Baseline						
	HS1						
	HS2						

a *It was not possible to compare pathways in the SPARE and IDM tools*.

b *The gray cells indicate that this pathway was not evaluated by the tool*.

c *In NORA, the human travel pathway had the highest risk for Finland in all three scenarios; the animal products pathway ranked second*.

d *Equal contribution of several pathways in one scenario*.

Overall, most tools agreed that (when considered) animal products constituted the highest risk to Finland for all three scenarios, and to the Netherlands in the baseline scenario. For both hypothetical scenarios the animal products pathway was still predicted to have the highest risk to the Netherlands by COMPARE and RRAT. However, NORA indicated a change in risk such that trade in live animals became the pathway with the highest risk for ASF incursion when the disease was assumed to be present in Germany. Under all scenarios the SVARRA tool did not have a single pathway constituting the highest risk to the Netherlands resulting from the lack of resolution available with a qualitative approach when risk estimates are close. The presence of ASF in Germany resulted in an increased risk estimate for the wild boar pathway in SVARRA, such that all three pathways had an equal risk level in the hypothetical scenarios. MINTRISK was the only tool not to consider animal products and concluded that from those that were included (trade in live animals and wild boar movements), wild boar was the pathway associated with the highest risk, for both the Netherlands and Finland.

Some of the tools included more pathways than the three investigated here (Table 2). When taking into account these additional pathways, only in NORA the pathway ranking top for Finland was changed from trade in animal products to human travel for all three scenarios (Supplementary Material 3).

## Discussion

### Cross-Validation Based on the ASF Case Study

Validation of generic RA tools is a challenging task for which no gold standard is available. In this study, we explored the opportunity to cross-validate seven generic RA tools by universally applying them to a predefined case study on the incursion risk of ASF. Comparison of the absolute results from the tools was not possible for several reasons, including their differing objectives, endpoints, outputs, and risk pathways considered. However, by comparing relative risks it was possible to cross-validate the generic RA tools across the different pathways, between the two countries of interest (Netherlands and Finland) and between the three scenarios that were evaluated.

In general, the tools agreed on the ranking of the target countries for the pathways evaluated, although the magnitude of relative risks calculated differed widely (Figure 3). All of the tools estimated that the live animal trade pathway posed a (much) higher risk to the Netherlands than Finland in the baseline scenario (2017 situation). The large differences in risk relate to the extremely low or even negligible incursion risk of trade in live animals to Finland rather than to a high risk to the Netherlands (absolute results for each tool are provided in Supplementary Material 3). In 2017, only 300 live pigs were transported to Finland compared to 1.9 million pigs to the Netherlands (33). All of the tools which included the animal products pathway agreed that the risk of this pathway to the Netherlands was higher than or equal to Finland in the baseline scenario with the exception of SPARE. This might be explained by the relatively large amount of pork and pork products imported by Finland from Estonia (33) and SPARE being the only tool not considering exposure, first infection or establishment, having entry as an endpoint. Hence, these imports from Estonia, which is an ASF-infected country, contributed largely to the estimated incursion risk by SPARE. For the wild boar pathway, all of the tools including this pathway agreed that the risk to Finland was higher than or equal to the Netherlands, although differences in risks between the countries were less pronounced than for the live animal trade pathway. SPARE calculated a high relative risk of this pathway to Finland if compared to the other tools, which again might be explained by the fact that this tool had entry as an endpoint. A lot of uncertainty was included in the predictions for the wild boar pathway, especially in the estimated risk to Finland, due to uncertainty on the presence of wild boar in the border region of Finland and Russia and the spatial distribution of ASF cases in wild boar in Russia.

When comparing the hypothetical scenarios to the baseline all of the RA tools indicated an increased risk to the Netherlands due to presence of ASF in wild boar and/or domestic pigs in Germany, especially for the wild boar pathway, whereas most tools agreed that the risk to Finland would stay at the same level or increase slightly. However, differences were observed between the tools on the extent to which the live animal trade pathway contributed to the increased risk to the Netherlands.

Investigating the ranking of risk pathways, comparisons could only be made for five of the seven tools, as the risk estimates for individual pathways in SPARE and IDM were given in different units and at a different scale, respectively. Comparisons of rankings were further complicated by the different numbers and types of pathways evaluated by each of the tools. Although Table 4 indicates that most tools agreed that the animal products pathway constituted the highest risk to both countries in the baseline scenario, this is actually only true for three out of the five tools that could be compared (COMPARE, RRAT, and SVARRA), with MINTRISK not having evaluated this pathway and NORA estimating a higher risk for the human travel pathway for Finland (Supplementary Material 3). In NORA, the human travel pathway includes the risk of ASF incursion via animal products carried for own consumption. Bringing products from Estonia was assumed to be common practice in Finland due to easy accessibility of wild boar products in Estonia at low prices, contributing largely to the high estimate of the risk of the human travel pathway to Finland. Lastly, it should be noted that SVARRA evaluated the risk of the live animal trade pathway equal to the animal products pathway for the Netherlands.

Differences in calculated relative risks over countries and scenarios between the tools can be largely explained from (i) differences in endpoints considered when evaluating the risk of ASF incursion, (ii) different quantitative scales on which the risk estimates were scored, and (iii) differences in the definition of pathways.

SPARE was the only tool that evaluated the entry of ASF into the target area without considering exposure or first infection of native animals. This has probably contributed to the differences observed between SPARE and the other RA tools when evaluating the relative risk from the animal products and the wild boar pathways to the Netherlands compared to Finland in the baseline scenario (Figure 3). No such differences between SPARE and the other tools were observed for the live animal trade pathway, where entry is more directly linked to exposure, first infection and establishment if animals are imported for life.

All tools but SVARRA produced numbers to express the incursion risk of ASF to the Netherlands and Finland, although the quantitative scales differed among the tools (see Table 3). Absolute results from SPARE and MINTRISK could in theory run from zero to infinity, even though most estimates were below 1 given the relatively low risk of disease incursion. Again, as SPARE evaluated entry rather than exposure, first infection or establishment, its results were relatively high for the animal products pathway (Supplementary Material 3). Absolute results from COMPARE, RRAT and NORA were bounded by 0 and 1. Thus, for all these five tools, whether absolute results were given from 0 to 1 or from 0 to infinity, calculated relative risks could run from zero to infinity and some tools indeed had extremely high relative risk scores (Figures 3, 4), especially if the denominator was estimated to be negligible or zero. Results from IDM, on the contrary, are semi-quantitative risk scores ranging from 0 to 60 for the overall risk, with maximum scores for individual pathways being < 60. As risk scores of IDM are discrete values (at an interval of 0.5), calculated relative risks were in general relatively low compared to those from other tools. Qualitative probability levels from SVARRA were converted into quantitative numbers using a log10 scale. Although this would have allowed the calculated relative risks to vary from 10^−4^ to 10^4^ with a scale consisting of five levels (ranging from very low to very high), the relative risks estimated by SVARRA never exceeded 10, since differences in risk were always equal to or less than one probability level.

Differences in calculated relative risks over countries and scenarios might also have originated from how pathways were defined in the tools. Trade in live animals was most uniformly defined, although some tools (SPARE, RRAT, IDM) not only included trade in domestic livestock animals, but also trade in horses, pets, and exotic mammals and birds. This will, however, not have resulted in differences for the ASF risk, since the only animals affected by ASF are porcine species (including wild boar and warthogs) (21, 22, 59, 62). Trade in animal products differed to a larger extent, with some tools only including pork and pork products for human consumption (SPARE, COMPARE), whereas other tools also included other products derived from slaughtered pigs, such as hides and bristles (RRAT, IDM). In IDM, NORA and SVARRA, this pathway even included illegal trade, whereas that was separated out in the other tools. The different definitions of the animal products pathway might have lead to different estimates of the absolute incursion risk of this pathway by the different tools, especially from source countries from which imports of fresh and frozen meat are not allowed. Also when considering the relative risks, the generic tools agreed least on the animal products pathway (Figure 3). Although the wild boar pathway was uniformly defined among the tools to be the incursion of ASF due to wild boar movements, this pathway was modeled differently among the tools, with some tools estimating the risk based on geographic proximity of infected wild boar populations at the country level (SPARE, MINTRISK, IDM), whereas one tool modeled the spatial distribution of wild boar and their movements based on habitat suitability (COMPARE).

MINTRISK is the only tool in which pathways are not predefined. The risk assessor can thus decide upon the pathways to include in the risk assessment and will preferably include those that are deemed most important given the transmission mechanisms of the disease. Although trade in animal products was considered an important route for ASF, the risk assessors decided not to include it because of lack of information on whether animal products were derived from domestic pigs or wild boar. The other tools did not offer the opportunity to distinguish between products from domestic pigs and wild boar and included both in the animal products pathway. The asset of having user-defined pathways in MINTRISK thus created another level of uncertainty in the results of the risk assessment which is beyond parameter and model uncertainty.

Differences in (implicit) modeling assumptions between the generic RA tools might have further contributed to the observed differences in the evaluated ASF incursion risk between the tools, although it is difficult to predict their impact on the calculated relative risks. For instance, the level of detail at which contact with susceptible animals in the target area was modeled varied widely among the tools, with some of them only using an overall probability estimate (RRAT, MINTRISK, IDM, NORA), while others explicitly modeled how different animal species (COMPARE) or livestock farms (SVARRA) would be exposed. Another example is that RRAT and NORA used worst case assumptions to evaluate the ASF incursion risk whereas the other tools used more realistic assumptions. Furthermore, the time period for which the risk assessment was performed differed among the tools. Most tools used an annual timescale and thus assessed the ASF incursion risk using data from a 1-year period (here: 2017). However, NORA and SVARRA considered a 3-month period to account for the high-risk period of newly infected territories, when no trade restrictions are in force yet. For some tools, decisions on these issues were embedded in the model structure, and could thus be attributed to model uncertainty, whereas for other tools, decisions were taken by the risk assessor performing the ASF case study.

The ultimate aim of these generic RA tools is to provide risk-based evidence to support risk managers in making informed decisions on reducing the incursion risk of infectious animal diseases by, e.g., preventive actions, targeted surveillance, and contingency planning. While absolute risk estimates contribute to the decision on whether any preventive actions are required or not, relative risk estimates are useful for prioritization of risk management options. Prioritization of diseases, pathways and/or target areas is an important output of all seven generic RA tools in this study (Table 1). Comparing the results of the tools for the ASF case study indicated that the tools largely agreed upon the direction of the relative risks and thus on prioritization of countries and scenarios. All tools concluded that the ASF risk of trade in live animals was lower to Finland than the Netherlands in the baseline scenario (2017), and that the risk of wild boar movements to Finland was equal to or higher than the Netherlands. Furthermore, all tools concluded that the presence of ASF in Germany (hypothetical scenarios) had little or no impact on the ASF risk to Finland, but did increase the ASF risk to the Netherlands. Thus, we concluded that the cross-validation contributed to the credibility of the results obtained with the generic RA tools.

Further validation of generic RA tools could be achieved by comparing their outcome for a specific risk question with results from bespoke models that were specifically developed to address this risk question. Although several bespoke models were developed for ASF in recent years (26–31), results of these models could not be used directly for comparison purposes as they addressed the ASF incursion risk for different years and/or countries than we did. Updating, re-parameterizing and re-running these models for the ASF case study was beyond the scope of this study. Two bespoke models were, however, developed that addressed the same risk question as the generic RA tools, one model assessing the probability of a first ASF infection in a new territory resulting from trade in live animals and the other model assessing the probability of entry of ASF virus in a new territory due to wild boar movements (63). Both models were parameterized to assess the ASF risk to the Netherlands and Finland in the 2017 situation and for the two hypothetical scenarios with ASF present in Germany. Results for trade in live animals indicated a higher risk to the Netherlands than Finland in the baseline scenario, with the calculated relative risk being in the same order of magnitude as those of the generic RA tools (Supplementary Material 4). The bespoke model for trade in live animals predicted an increased risk only to the Netherlands for HS2, i.e., when ASF cases were also reported in domestic pigs, with a calculated relative risk just above 1 if compared to the baseline scenario. Again, this is in agreement with the results from the generic RA tools (Supplementary Material 4). Results of the bespoke model for the wild boar pathway indicated an equally negligible risk to the Netherlands and Finland in the baseline scenario. In HS1, the risk of this pathway was increased to the Netherlands with no further increase in HS2. The hypothetical scenarios did not result in an increased risk to Finland for the wild boar pathway. These results are in agreement with results from the generic RA tools, with none of them predicting an increased risk to Finland for the wild boar pathway under the hypothetical scenarios and all of them but one predicting an equally increased risk under HS1 and HS2 to the Netherlands (Supplementary Material 4).

Application of the tools for a prolonged period might create an opportunity for external validation using field data. Some of the generic tools have been up and running for at least 5 years now. IDM was first released in 2011 and has intensively been used in the UK by Defra and the Scottish Government's Center of Expertise on Animal Disease Outbreaks (EPIC) to prioritize their risk levels for incursion of disease at different times of the year. SVARRA was first used in 2013 and has been used for several rapid risk assessments including ASF, BT, LSD, and avian influenza (AI). However, one of the difficulties in validating models evaluating the incursion risk of exotic diseases is that the adverse events being modeled have a low probability of occurrence resulting in too few data points for validation, even when used for a prolonged period.

### Model Uncertainty

This study clearly illustrates the impact of model uncertainty on risk assessment results. Although the generic RA tools agreed on the direction of the relative risk of ASF to the Netherlands and Finland, the magnitudes of these relative risks varied largely, especially for the live animal trade and wild boar pathways. The range of results obtained when considering the results of all generic RA tools could be interpreted as an indication of the uncertainty included in the risk estimates. The results of the bespoke models fell well within this uncertainty range.

Several methods exist to combine the results of different models that predict similar metrics, such as ensemble modeling, structured decision making and model averaging (64). Although widely applied in, for example, weather prediction (65), ensemble modeling is still at its infancy in veterinary epidemiology (64, 66). Methods like structured decision making and ensemble modeling can only be applied to models that produce similar metrics to compare results accross models. It was thus not possible to combine the model outputs of the generic RA tools involved in this study to produce an uncertainty distribution of the modeled ASF risk as output parameters represented different endpoints, varied from qualitative to quantitative, and were expressed in different units. To make an integrated risk estimate from the generic RA tools possible, output parameters would need to be harmonized first. A further impediment to the integration of risk estimates obtained by the generic RA tools is the difference in pathways evaluated by each of the tools. Nevertheless, a mere comparison of results of different tools can already be helpful in obtaining a more complete picture of the risk and the uncertainties involved as illustrated by this study.

The choice for one or more generic RA tools to answer a specific risk question will depend on the primary objective of the risk assessment, the diseases and pathways that need to be evaluated, the resources and expertise available, and the timescale at which the risk assessment has to be completed (see Tables 1, 2 and Supplementary Material 5). Some of the tools allow for a rapid risk assessment in response to a new disease event (NORA, SVARRA), whereas others can be used for a continuous assessment of incursion risks over time making them suitable for horizon scanning (SPARE, COMPARE, RRAT, IDM). The latter tools could, for instance, be used to monitor the incursion risk of ASF for a specific target area by comparing results obtained for previous years with the current situation. Some of the tools come with a prefilled database for specific diseases and pathways and can easily be used to perform updates of risk assessments for these diseases (SPARE, COMPARE, RRAT, IDM), whereas other tools do not come with an underlying database and have to be filled by the risk assessor (MINTRISK, NORA, SVARRA). For these tools, disease expertise is a prerequisite to perform the risk assessment, whereas some of the prefilled tools mainly require computing expertise. Generally speaking, SVARRA and MINTRISK are most flexible as to which pathways and diseases to include. SVARRA is very well-suited for rapid risk assessments in response to disease events such as the incursion risk of ASF in wild boar in Belgium in September 2018 (2). MINTRISK was primarily developed to evaluate the incursion risk of vector-borne diseases. Although ASF can be transmitted by soft ticks of the genus *Ornithodoros*, these vectors do not seem to play a role in ASF virus transmission in Europe (23). Hence, MINTRISK is not a preferred tool to evaluate the risk of ASF. Some of the generic RA tools can rapidly assess the risk of multiple diseases for a target area given the diseases have been included in the tool (SPARE, COMPARE, RRAT, IDM). Results of these tools can be used to evaluate the relative risk of ASF compared to the risk of other notifiable diseases that might threaten the target area on a regular basis. Most of the generic RA tools can break down results to provide more detail on source areas and pathways contributing most to the risk or to indicate regions in the target area most at risk of incursion of a new disease, all of which is essential information for disease prevention and surveillance purposes. Communication of results from generic RA tools to risk managers should therefore aim at a proper understanding of the risks and the uncertainties involved by indicating underlying mechanisms rather than at communicating the absolute value or level of risk predicted.

## Data Availability Statement

All datasets generated for this study are included in the article/Supplementary Material.

## Author Contributions

CV and ES initiated and conceptualized the research. RS provided harmonized data from Eurostat and WAHIS. RS performed the case study in SPARE. RT and ES performed the case study in COMPARE. AK, MC, and AB performed the case study in MINTRISK. RP, MS, and CV performed the case study in RRAT. HR performed the case study in IDM. TL and LS-L performed the case study in NORA. CH, KS, and AC performed the case study in SVARRA. SN developed the bespoke models to evaluate the ASF incursion risk. CV did the analysis of results and drafted the manuscript. All authors reviewed and edited the manuscript.

### Conflict of Interest

The authors declare that the research was conducted in the absence of any commercial or financial relationships that could be construed as a potential conflict of interest.

## Footnotes

^1^Generic approaches for Risk Assessment of Infectious animal Disease introduction.
