# Supplementary material for: Cross-Validation of Generic Risk Assessment Tools for Animal Disease Incursion Based on a Case Study for African Swine Fever
Source: Front Vet Sci. 2020 Feb 18;7:56. doi: 10.3389/fvets.2020.00056 (PMC7039936; doi:10.3389/fvets.2020.00056)
Supplement: Supplementary file 1 [file Table_1.docx]

Supplementary Material 1: Description of generic risk assessment tools

# SPARE: Development of SPatial risk assessment framework for Assessing exotic disease incuRsion through Europe

- To develop an overarching model to make rapid use of available metadata to identify pathways of potential risk for classes of disease transmission (e.g. vector-borne).
- Objectives: To provide valuable information for risk assessors in the first instances of a disease outbreak where typically information on imports and routes of entry and potential for spread is undertaken on a case-by-case basis. It will also allow for an objective and systematic evaluation to inform risk-based animal health and zoonotic surveillance activities.
- Pathways: Legal trade in livestock and pets, legal and illegal trade in products of animal origin, human travel by aircraft, vector dispersion, wild animal dispersion.
- Diseases: The case studies in the original project were classical swine fever (CSF), classical rabies and bluetongue.
- Main elements: The main routes of transmission considered are; Live animals, trade of meat products, wild animal dispersion, windborne spread of vectors, people movement and pets.
- Main inputs:
  - Country level pathogen prevalence/numbers (OIE, WAHIS annual/weekly reports)
  - Volumes of trade between countries (Comext, Eurostat)
  - Human travel data (Eurostat)
  - Pet movement data (TRACES)
  - Livestock density maps (FAO)
  - Wild animal prevalence/density/habitat suitability maps (from wider SPARE project or published literature)
- Main algorithms:
  - Algorithms to obtain and format input data from the internet
  - Distributions for expected number of cases per country
  - Spatial algorithms to assess expected dispersion of vectors/wild animals
  - Expected number and/or probability of introduction into EU MSs for each route/pathogen/country combination e.g. expected number of live animals infected with CSF imported to Spain from Brazil, probability of at least one mosquito infected with bluetongue entering Italy from Tunisia.
- Outputs:
  - Relative risk scores to rank diseases, pathways and/or areas of origin.
  - User friendly visualization interface, to present outputs in the form of maps.
  - Results from various stages of the model (e.g. distributions for expected number of cases, expected number and/or probability of introduction into EU MSs) can be used as inputs for a subsequent exposure assessment.
- Main author: Robin Simons, APHA, United Kingdom.
- Software: R
- Release and upgrades: Final version 2018.
- More information: Simons et al., 2017; Simons et al., 2019.

# COMPARE: COllaborative Management Platform for detection and Analyses of (Re-) emerging and foodborne outbreaks in Europe

- Framework and R code that allows calculation of risk of infection for any disease from any area to any other area at any spatial scale, depending on data availability.
- Objectives: To identify hotspots for infection to target surveillance; to allow comparisons across diseases and pathways.
- Pathways: Legal trade of live animals, legal trade of food products, wild animal movement, vector dispersion, human travel, bird migration.
- Diseases: The completed case studies are lumpy skin disease, African swine fever and Zika. A case study for avian influenza is also planned.
- Main elements: Risk of infection at different spatial scales; risk of spreading to new areas.
- Main input: Prevalence data (derived from SPARE or calculated using Empres-i data), trade data (Comext and TRACES), abundance/presence of wild animals, number of farms and animals in area of interest, disease-related parameters.
- Main algorithms: Calculation of probability of first infection and spread based on modelling, quantitative input and stochastic simulations to produce a distribution of risk. Different models included for different pathways, with the main pathways being trade of livestock and products, vector-borne, wild animal introduction, migratory birds and human travel.
- Output: Color-coded maps at different spatial scales showing the risk of infection and spread in different areas, with separate maps showing variability.
- Main author: Rachel Taylor, APHA, United Kingdom.
- Software: R
- Release and upgrades: First release: September 2018.
- More information: Taylor et al., 2018; Taylor et al., 2019a; Taylor et al., 2019b; Taylor et al., 2019c.

# RRAT: Rapid Risk Assessment Tool

- Relational database that can be queried to answer risk questions related to the introduction of exotic animal diseases.
- Objectives: To prioritize diseases for risk management and early warning; to identify high risk trade flows.
- Pathways: Legal trade in live animals, germplasm and products of animal origin.
- Diseases: The model has been parameterized to estimate the risk for ten diseases, viz. African swine fever, bluetongue, equine infectious anemia, lumpy skin disease, peste des petits ruminants, bovine tuberculosis, pseudorabies, classical swine fever, foot and mouth disease, and African horse sickness. Avian influenza will be included in a later stage.
- Main elements: Probability of entry, probability of establishment.
- Main input: Tables on worldwide distribution of diseases (WAHIS), on trade in animals and animal products and other pathways (TRACES, Comext Eurostat), and on infectivity of pathways such as susceptibility of animal species, disease parameters, and contamination of animal products (information derived from literature).
- Main algorithms: Calculation of probability of introduction and establishment of diseases into the Netherlands; while pathway numbers are based on quantitative data, probabilities of infection and establishment are based on semi-quantitative values derived from risk categories.
- Output: Relative risk scores to rank diseases, pathways and/or areas of origin.
- Main author: Clazien de Vos, WBVR, The Netherlands
- Software: R and SQLite.
- Release and upgrades: First version to be finished in 2020.
- More information: De Vos et al., 2018.

# MINTRISK: Method for INTegrated RISK assessment of vector-borne diseases

- Web-based calculation tool for quick and more in-depth risk assessments of vector-borne diseases.
- Objectives: To ensure completeness and consistency in risk assessment of vector-borne diseases; for comparison and prioritization.
- Pathways: User-defined.
- Diseases: Vector-borne diseases, user-defined.
- Main elements: Probability of entry, transmission, and establishment, extent of spread, likelihood of persistence, impact.
- Main input: Answering of questions that have qualitative answer categories with a quantitative explanation.
- Main algorithms: Sampling from triangular distributions on a linear scale between 0 and 1; the sampling range is based on the selected answer category and indicated uncertainty; the sampled risk scores are log-transformed into quantitative values that are consistent with the quantitative explanation of the answer category chosen; input values are combined using mathematical algorithms that are based on the principles of risk assessment and mathematical modelling in epidemiology; output values are back-transformed into a risk score, where the relevant output levels are scaled between 0 and 1.
- Output: Risk scores between 0 and 1 translated into qualitative risk scores for each step in the model and for the overall risk estimate; two additional summarizing risk scores are provided: rate of introduction and epidemic size; use of a two-dimensional risk profile diagram indicating the overall probability of introduction versus the epidemic size or the impact of disease.
- Main author: Aline de Koeijer, WBVR, The Netherlands.
- Software: Visual Studio with C# for algorithms.
- Release and upgrades: First version in Excel and Visual Basic in 2014; upgrade to web-based version in 2015; upgrade expected in 2020; special version for EFSA in 2015.
- More information: De Vos et al., 2012; De Vos et al., 2016; EFSA AHAW Panel, 2017; EFSA 2017.

# IDM: International Disease Monitoring tool for risk of incursion

- A semi-quantitative rapid risk assessment tool for incursion of disease through trade routes and other high risk pathways .
- Objectives: To provide an evidence base for the most high priority exotic notifiable diseases and the risk of incursion for the UK. The tool compares different pathways (trade of live animals, products and germinal products, vectors, wildlife and transport) for different diseases to give a comparative risk score and help policy makers decide on areas for concern.
- Pathways: Trade in live animals including horses, pets, and the Balai animals, trade in products of animal origin (POAO), transportation, and migratory birds.
- Diseases: The model includes a large number of mostly exotic notifiable diseases, but can do any new or emerging disease.
- Main elements: Excel workbook with interlinked spreadsheets giving scores for the level of trade from and the presence of disease in different geographic regions (not individual countries) for different diseases. Mitigation is based on whether regulations or disease control measures are in place at the country of origin. A final additive risk score for each pathway is then compared between all the diseases, graphically.
- Main input: Disease presence in wildlife or domestic livestock (either as sporadic domestic outbreaks in small non-commercial farms or widespread in commercial farms); trade volume as a score of 0 to 3 for geographic regions and for different risk products (live animals, germinal products, products of animal origin); option for including transport (fomites), vectors and exotic animal trade; a “degree of separation” score is used to account for whether there are overlapping wild bird migration routes and the same proximity score is used for wildlife movements in the ASF case study.
- Main algorithms: Risk scores are calculated by multiplying the scores for the pathways and trade volume, adding the risk score for additional pathways (migratory birds) and by subtracting the risk score for mitigation.
- Output: Table and graph comparing the diseases.
- Main author: Helen Roberts, APHA, United Kingdom.
- Software: Excel
- Release and upgrades: First release: 2011. Regular updates as requests were made to include new diseases. Updated seasonally or if a large jump in disease distribution is seen. New version in 2013 to account for wild bird migration routes.
- More information: Roberts et al., 2011; EFSA, 2017.

# NORA: Nopea riskinarviointityökalu (rapid risk assessment tool)

- NORA was developed for situations where there is a change in the disease status of easily transmissible animal diseases in neighboring countries or in countries with significant interactions with Finland.
- Objectives: The goal was to develop a tool that is quick to use and will provide consistent results in order to support risk management decisions.
- Pathways: Trade in live animals, germplasm and products of animal origin, vector dispersion, wild animal dispersion, airborne spread, feed and bedding, and fomites and transport means related to human travel.
- Diseases: NORA has been parameterized for African swine fever, lumpy skin disease, chronic wasting disease, foot and mouth disease, and bluetongue.
- Main elements: importance/relevance of different introduction routes, probability score of entry and establishment (in other words introduction), impact.
- Main input: Answering of questions that have qualitative answer categories with a quantitative explanation.
- Main algorithms: Combination of values within a route are calculated by applying the basic probability calculation rules of serial (multiplying) and parallel (summing) processes. There are no Monte Carlo simulations in the estimation.
- Output: The potentiality of nine different introduction routes, their relative importance and the overall risk of introduction on a semi-quantitative scale. If the impact score is assessed, there is a possibility to also estimate combined risk that is relative to FMD impact of Finnish RA.
- Main author: Tapani Lyytikäinen, Ruokavirasto, Finland.
- Software: Excel
- Release and upgrades: First version in 2015 in Finnish; English version in 2018.
- More information: Kyyrö et al., 2017.

# SVARRA: Rapid risk assessment tool for introduction of exotic disease to the Swedish animal population

- Qualitative risk assessment tool to assess the probability of introduction of exotic diseases into the Swedish animal population.
- Objectives: To ensure systematic, structured, transparent and well documented qualitative rapid risk assessments.
- Pathways: Live animals, germplasm (semen, ova, embryos), indirect transmission (vehicles, persons including clothes and equipment, feed and bedding), animal products, vectors and wild animals.
- Diseases: Designed to be used for any disease. The SVARRA has so far been used for risk assessments on avian influenza, African swine fever, lumpy skin disease, peste des petits ruminants, and bluetongue.
- Main elements: Probability of entry, probability of exposure. The SVARRA consists of three parts: instructions, a template to be filled out by the risk assessor, and a summarizing report based on the template. Only the summarizing report is communicated to external parties. The template provides the documentation of the risk assessment.
- Main input: Answering of probability questions related to pre-established risk pathways with answers being based on import data, legal requirements in country of origin and Sweden, expert opinion on illegal trade, indirect transmission, and wildlife populations. Uncertainty is assessed for each probability question.
- Main algorithms: Risk matrix to combine the estimated probabilities of entry and exposure per pathway.
- Output: Probability of exposure of Swedish wild or domesticated animal populations expressed in qualitative terminology: negligible, very low, low, medium, high, very high. Uncertainty is expressed using the terms low-medium-high. Both sets of terms are provided with an interpretation of each term. Result of the assessment is communicated in a 3-page report.
- Main authors: Cecilia Hultén and Kaisa Sörén, SVA, Sweden.
- Software: Word (2013 version); Excel (2018 version; developed by Arianna Comin)
- Release and upgrades: First (Swedish) version in Word in 2013; English version in Word in 2018; Excel version in 2018.
- More information: EFSA, 2017.

# References

De Vos C, Hoek M, Fischer E, De Koeijer A, Bremmer J, 2012. Risk assessment framework for emerging vector-borne livestock diseases. Report 11-CVI0168. Available from: <http://edepot.wur.nl/198115>. Last accessed 4 June 2019.

De Vos CJ, Van Roermund HJW, De Koeijer AA, Fischer EAJ, 2016. Risk assessment of seven emerging vector-borne animal diseases for The Netherlands: A structured approach. In: Lindberg A, Thulke HH, editors. Society for Veterinary Epidemiology and Preventive Medicine. Proceedings of a meeting held in Elsinore, Denmark, 16^th^-18^th^ March 2016. p. 215-228.

De Vos CJ, Petie R, Van Klink E, Swanenburg M, 2018. Rapid risk assessment of exotic animal disease introduction. The 15^th^ International Symposium of Veterinary Epidemiology and Economics. 12-16 November 2018, Chiang Mai, Thailand. p. 253.

EFSA, 2017. EFSA webinar: rapid risk assessment tools for animal disease outbreaks. 27 November 2017. Available from: <http://www.efsa.europa.eu/en/events/event/171127>. Last accessed 6 March 2019.

EFSA AHAW Panel, 2017. Scientific opinion on vector-borne diseases. *EFSA Journal* 15(5):4793. doi:10.2903/j.efsa.2017.4793

Kyyrö J, Sahlström L, Lyytikäinen T, 2017. Assessment of the risk of African swine fever introduction into Finland using NORA – a rapid tool for semiquantitative assessment of the risk. *Transbound Emerg Dis* 64:2113-2125. doi:10.1111/tbed.12633

Roberts H, Carbon M, Hartley M, Sabirovic M, 2011. Assessing the risk of disease introduction in imports. *Vet Rec* 168:447-448. doi:10.1136/vr.d1784

Simons RRL, Horigan V, De Nardi M, Ru G, Pena AE, Adkin A. Mighty models from little data grow: Estimating animal disease prevalence. In: Nielsen LR, Lindberg A, editors. Society for Veterinary Epidemiology and Preventive Medicine. Proceedings of a meeting held in Inverness, Scotland, UK, 29^th^ -31^st^ March 2017 (2017). p. 166-175.

Simons RRL, Horigan V, Ip S, Taylor RA, Crescio MI, Maurella C, et al., 2019. A spatial risk assessment model framework for incursion of exotic animal disease into the European Union Member States. *Microbial Risk Anal* 13:100075. doi:10.1016/j.mran.2019.05.001

Taylor RA, Gale P, Kelly LA, Snary EL, 2018. One for all: a generic risk framework for all pathways of disease introduction. The 15^th^ International Symposium of Veterinary Epidemiology and Economics. 12-16 November 2018, Chiang Mai, Thailand. p. 650.

Taylor RA, Berriman ADC, Gale P, Kelly LA, Snary EL, 2019a. A generic framework for spatial quantitative risk assessments of infectious diseases: Lumpy skin disease case study. *Transbound Emer. Dis* 66(1):131-143. doi:10.1111/tbed.12993

Taylor RA, Podgórski T, Simons RRL, Ip S, Gale P, Kelly LA, et al., 2019b Predicting spread and effective control measures for African swine fever– should we blame the boars? *bioRxiv* 654160. doi:10.1101/654160

Taylor RA, Condoleo R, Simons RRL, Gale P, Kelly LA, Snary EL. The Risk of Infection by African Swine Fever Virus in European Swine Through Boar Movement and Legal Trade of Pigs and Pig Meat *Front Vet Sci* (2020) 6:486. doi:10.3389/fvets.2019.00486
