# Supplementary material for: Cross-Validation of Generic Risk Assessment Tools for Animal Disease Incursion Based on a Case Study for African Swine Fever
Source: Front Vet Sci. 2020 Feb 18;7:56. doi: 10.3389/fvets.2020.00056 (PMC7039936; doi:10.3389/fvets.2020.00056)
Supplement: Supplementary file 2 [file Table_2.DOCX]

Supplementary Material 2: Harmonized input data used by the seven generic risk assessment tools for the African swine fever case study

Table 1. Data used to estimate African swine fever (ASF) prevalence in source countries: ASF cases in domestic pigs and wild boar reported to OIE in 2017, and domestic pig populations in ASF-infected countries (OIE, 2018a).

| Country | ASF cases in domestic pigs | ASF cases in wild boar | Domestic pig population |
| --- | --- | --- | --- |

| Czech Republic | 0 | 185 | 1282007 |
| --- | --- | --- | --- |
| Estonia | 3 | 637 | 289972 |
| Italy^a^ | 28 | 96 | 12050938 |
| Ivory Coast | 3440 | 0 | 211826 |
| Latvia | 272 | 1051 | 327205 |
| Lithuania | 67 | 614 | 622803 |
| Moldova | 31 | 3 | 619110 |
| Poland | 405 | 964 | 14037301 |
| Romania | 1 | 0 | 5362449 |
| Russia | 2210 | 169 | 22027698^b^ |
| Ukraine | 1212 | 44 | 5995739 |
| South Africa | 272 | 0 | 1480000 |
| Zambia | 1174 | 0 | 1166055^b^ |

^a^ Cases reported for Italy were derived from ADNS (EC, 2018). These were all cases in Sardinia which has consistently had cases for a long time without observed spillover to the rest of Italy or Europe as a whole. To avoid overestimating the risk of spread from Italy, these cases were omitted from the analyses.

^b^ No data available from OIE (2018a). Estimates based on data from FAO (2018).

Table 2. Trade volumes of live pigs and pig products for the Netherlands (NLD) and Finland (FIN) in 2017 (Eurostat, 2018).

| Source Country | Live pigs^a^ (numbers) | | Pork^b^ (kg) | | Pork products^c^ (kg) | |
| --- | --- | --- | --- | --- | --- | --- |
|  | NLD | FIN | NLD | FIN | NLD | FIN |
| Argentina | 0 | 0 | 8 | 0 | 0 | 0 |
| Australia | 0 | 0 | 216288 | 0 | 0 | 0 |
| Austria | 9289 | 0 | 1092485 | 730994 | 119569 | 167306 |
| Bangladesh | 0 | 0 | 16 | 0 | 0 | 0 |
| Belarus | 0 | 0 | 3 | 0 | 0 | 0 |
| Belgium | 976428 | 0 | 40784516 | 99150 | 1429950 | 0 |
| Brazil | 0 | 0 | 24 | 0 | 0 | 0 |
| Bulgaria | 384 | 0 | 128330 | 0 | 11402 | 0 |
| Cambodia | 0 | 0 | 1 | 0 | 0 | 0 |
| Canada | 18 | 0 | 17 | 0 | 0 | 0 |
| Chile | 0 | 0 | 4 | 0 | 0 | 0 |
| China | 0 | 0 | 267 | 0 | 0 | 0 |
| Colombia | 0 | 0 | 1 | 0 | 0 | 0 |
| Costa Rica | 0 | 0 | 9 | 0 | 0 | 0 |
| Croatia | 43062 | 0 | 39468 | 0 | 1613 | 0 |
| Cuba | 0 | 0 | 2 | 0 | 0 | 0 |
| Cyprus | 59 | 0 | 88968 | 0 | 681 | 0 |
| Czech Republic | 10966 | 0 | 360777 | 0 | 19958 | 0 |
| Denmark | 276176 | 254 | 9740880 | 1403570 | 57120 | 937692 |
| Ecuador | 0 | 0 | 7 | 0 | 0 | 0 |
| Egypt | 0 | 0 | 4 | 0 | 0 | 0 |
| Estonia | 1258 | 0 | 31768 | 776629 | 1751 | 282 |
| Ethiopia | 0 | 0 | 4 | 0 | 0 | 0 |
| Finland | 1433 | 0 | 161248 | 0 | 9488 | 0 |
| France | 53827 | 0 | 15921868 | 3901 | 190961 | 31324 |
| Gabon | 0 | 0 | 1 | 0 | 0 | 0 |
| Germany | 361966 | 0 | 186083047 | 12987349 | 4596647 | 361497 |
| Greece | 486 | 0 | 143324 | 0 | 6345 | 0 |
| Guatemala | 0 | 0 | 1 | 0 | 0 | 0 |
| Hong Kong | 0 | 0 | 26 | 0 | 0 | 0 |
| Hungary | 81815 | 0 | 1426837 | 339919 | 10804 | 4465 |
| Iceland | 0 | 0 | 4 | 0 | 0 | 0 |
| India | 0 | 0 | 43 | 0 | 0 | 0 |
| Indonesia | 0 | 0 | 19 | 0 | 0 | 0 |
| Iran | 0 | 0 | 1 | 0 | 0 | 0 |
| Ireland | 7933 | 0 | 2803567 | 228 | 58093 | 0 |
| Source Country | Live pigs^a^ (numbers) | | Pork^b^ (kg) | | Pork products^c^ (kg) | |
|  | NLD | FIN | NLD | FIN | NLD | FIN |
| Israel | 0 | 0 | 12 | 0 | 0 | 0 |
| Italy | 16174 | 0 | 4570981 | 4574 | 1189877 | 175614 |
| Japan | 0 | 0 | 73 | 0 | 0 | 0 |
| Jordan | 0 | 0 | 1 | 0 | 0 | 0 |
| Kenya | 0 | 0 | 11 | 0 | 0 | 0 |
| Latvia | 1060 | 0 | 80468 | 0 | 4190 | 0 |
| Lebanon | 0 | 0 | 1 | 0 | 0 | 0 |
| Libya | 0 | 0 | 1 | 0 | 0 | 0 |
| Lithuania | 726 | 0 | 180544 | 0 | 4647 | 0 |
| Luxembourg | 6468 | 0 | 96286 | 0 | 30685 | 0 |
| Malaysia | 0 | 0 | 16 | 0 | 0 | 0 |
| Malta | 23 | 0 | 5075 | 0 | 280 | 0 |
| Mauritius | 0 | 0 | 1 | 0 | 0 | 0 |
| Mexico | 0 | 0 | 3 | 0 | 0 | 0 |
| Morocco | 0 | 0 | 2 | 0 | 0 | 0 |
| Mozambique | 0 | 0 | 1 | 0 | 0 | 0 |
| Netherlands | 0 | 0 | 0 | 680136 | 0 | 272443 |
| New Zealand | 0 | 0 | 1 | 23999 | 0 | 0 |
| Norway | 394 | 46 | 2210 | 0 | 0 | 0 |
| Pakistan | 0 | 0 | 4 | 0 | 0 | 0 |
| Peru | 0 | 0 | 1 | 0 | 0 | 0 |
| Philippines | 0 | 0 | 3 | 0 | 0 | 0 |
| Poland | 63857 | 0 | 10949620 | 209499 | 246987 | 1664605 |
| Portugal | 1466 | 0 | 190889 | 0 | 13472 | 0 |
| Romania | 1336 | 0 | 127531 | 1638 | 16990 | 0 |
| Russia | 0 | 0 | 13 | 0 | 0 | 0 |
| Senegal | 0 | 0 | 1 | 0 | 0 | 0 |
| Singapore | 0 | 0 | 8 | 0 | 0 | 0 |
| Slovakia | 1855 | 0 | 1176988 | 0 | 8538 | 0 |
| Slovenia | 424 | 0 | 99796 | 0 | 3476 | 0 |
| South Africa | 0 | 0 | 27029 | 0 | 0 | 0 |
| South Korea | 0 | 0 | 12 | 0 | 0 | 0 |
| Spain | 9993 | 0 | 4283148 | 1856750 | 1044551 | 116718 |
| Sri Lanka | 0 | 0 | 1 | 0 | 0 | 0 |
| Sweden | 3261 | 0 | 3900312 | 171339 | 19089 | 492715 |
| Switzerland | 0 | 0 | 41 | 0 | 0 | 0 |
| Taiwan | 0 | 0 | 30 | 0 | 0 | 0 |
| Thailand | 0 | 0 | 16 | 0 | 0 | 0 |
| Turkey | 0 | 0 | 61 | 0 | 0 | 0 |
| Uganda | 0 | 0 | 1 | 0 | 0 | 0 |
| Ukraine | 0 | 0 | 7 | 0 | 0 | 0 |
| Source Country | Live pigs^a^ (numbers) | | Pork^b^ (kg) | | Pork products^c^ (kg) | |
|  | NLD | FIN | NLD | FIN | NLD | FIN |
| United Arab Emirates | 0 | 0 | 2 | 0 | 0 | 0 |
| United Kingdom | 14175 | 0 | 3939674 | 14000 | 173687 | 3850 |
| United States | 131 | 0 | 60991 | 0 | 0 | 0 |
| Uruguay | 0 | 0 | 2 | 0 | 0 | 0 |
| Vietnam | 0 | 0 | 18 | 0 | 0 | 1 |

^a^ All CN codes starting with 0103.

^b^ All CN codes starting with 0203.

^c^ CN codes 021011, 021012, 021019, 02109031, 02109039.

Table 3. Disease-related parameters for African swine fever used in the case study.

| \| Parameter \| Value \| Source \| \| --- \| --- \| --- \| \| Incubation period (days) \| 10.5 \| Average of multiple studies (Gulenkin et al., 2011; Blome et al., 2013; Sanchez-Vizcaino et al., 2015; Arias et al., 2018; OIE, 2018b) \| \| Infectious period (days) \| 7 \| Gabriel et al., 2011 \| \| Probability of transmission between wild boar and/or pigs \| 0.3 \| Pietschmann et al., 2015; Guinat et al., 2016 \| \| Probability of transmission from contact with a wild boar carcass \| 0.167 \| Pietschmann et al., 2015 \| \| Mortality rate \| 0.95 \| Gallardo et al., 2015; Thulke et al., 2017 \| \| ASF dose response rate \| 0.000012 \| Gale et al., 2004 \| \| R_0_ within-herd transmission \| 9 \| Gulenkin et al., 2011 \| \| R_0_ between-herd transmission \| 2.5 \| Gulenkin et al., 2011 \| |
| --- | --- | --- | --- | --- | --- | --- | --- | --- | --- | --- | --- | --- | --- | --- | --- | --- | --- | --- | --- | --- | --- | --- | --- | --- | --- | --- | --- |

# References

Arias M, Jurado C, Gallardo C, Fernández‐Pinero J, Sánchez‐Vizcaíno JM, 2018. Gaps in African swine fever: Analysis and priorities. *Transbound Emerg Dis* 65 Suppl 1:235-247. doi:10.1111/tbed.12695

Blome S, Gabriel C, Beer M, 2013. Pathogenesis of African swine fever in domestic pigs and European wild boar. *Virus Res* 173(1):122-130. doi:10.1016/j.virusres.2012.10.026

EC (European Commission), 2018. Animal Disease Notification System (ADNS). Available from: <https://ec.europa.eu/food/animals/animal-diseases/not-system_en>. Accessed in November 2018.

Eurostat, 2018. Comext bulk download. Available from: <https://ec.europa.eu/eurostat/estat-navtree-portlet-prod/BulkDownloadListing?sort=1&dir=comext>. Accessed in November 2018.

FAO, 2018. FAOSTAT. Data on live animals. Available from: <http://www.fao.org/faostat/en/#data/QA>. Accessed in November 2018.

Gabriel C, Blome S, Malogolovkin A, Parilov S, Kolbasov D, Teifke JP, Beer M, 2011. Characterization of African swine fever virus Caucasus isolate in European wild boars. *Emerg infect Dis* 17(12):2342. doi:10.3201/eid1712.110430

Gale P, 2004. Risks to farm animals from pathogens in composted catering waste containing meat. *Vet Rec* 155(3):77-82. doi:10.1136/vr.155.3.77

Gallardo MC, De la Torre Reoyo A, Fernández-Pinero J, Iglesias I, Muňoz MJ, Arias ML, 2015. African swine fever: a global view of the current challenge. *Porcine Health Management* 1:21. doi:10.1186/s40813-015-0013-y

Guinat C, Gubbins S, Vergne T, Gonzales JL, Dixon L, Pfeiffer DU, 2016. Experimental pig-to-pig transmission dynamics for African swine fever virus, Georgia 2007/1 strain. *Epidemiol Infect* 144(1): 25-34. doi:10.1017/S0950268815000862

Gulenkin VM, Korennoy FI, Karaulov AK, Dudnikov SA, 2011. Cartographical analysis of African swine fever outbreaks in the territory of the Russian Federation and computer modeling of the basic reproduction ratio. *Prev Vet Med* 102(3):167-174. doi:10.1016/j.prevetmed.2011.07.004

OIE. World Animal Health Information System (2018a). Available from: <http://www.oie.int/wahis_2/public/wahid.php/Wahidhome/Home/>. Accessed in November 2018.

OIE 2018b, African swine fever, OIE Technical Disease Card. Accessed in November 2018. <https://www.oie.int/fileadmin/Home/eng/Our_scientific_expertise/docs/pdf/AFRICAN%20SWINE%20FEVER.pdf>

Pietschmann J, Guinat C, Beer M, Pronin V, Tauscher K, Petrov A, Keil G, Blome S, 2015. Course and transmission characteristics of oral low-dose infection of domestic pigs and European wild boar with a Caucasian African swine fever virus isolate. *Arch Virol* 160(7):1657-1667. doi:10.1007/s00705-015-2430-2

Sánchez-Vizcaíno JM, Mur L, Bastos AD, Penrith ML, 2015. New insights into the role of ticks in African swine fever epidemiology. *Rev Sci Tech* 34(2):503-11.

Thulke HH, Lange M, 2017. Simulation‐based investigation of ASF spread and control in wildlife without consideration of human non‐compliance to biosecurity. *EFSA supporting publication* 2017:EN-1312. doi:10.2903/sp.efsa.2017.EN-1312
