# Supplementary material for: Cross-Validation of Generic Risk Assessment Tools for Animal Disease Incursion Based on a Case Study for African Swine Fever
Source: Front Vet Sci. 2020 Feb 18;7:56. doi: 10.3389/fvets.2020.00056 (PMC7039936; doi:10.3389/fvets.2020.00056)
Supplement: Supplementary file 3 [file Table_3.DOCX]

Supplementary Material 3: Results of the seven generic risk assessment tools included for the African swine fever study per scenario, country and pathway

Table 1. Calculated African swine fever incursion risk to the Netherlands (NLD) and Finland (FIN) in the 2017 situation (Base) and two hypothetical scenarios assuming African swine fever cases in Germany (HS1: wild boar only; HS2: wild boar and domestic pigs).

|  | **Results NLD** | | | **Results FIN** | | |
| --- | --- | --- | --- | --- | --- | --- |
|  | **Base** | **HS1** | **HS2** | **Base** | **HS1** | **HS2** |
| **SPARE (Entry; number of infected units per year)** |  |  |  |  |  |  |
| Live animals | 0.118 | 0.125 | 0.142 | 1.00×10^-9^ | 1.00×10^-9^ | 1.24×10^-9^ |
| Products of animal origin (kg) | 38.95 | 47.97 | 72.61 | 90.04 | 90.33 | 91.1215 |
| Wild boar | 1.85×10^-5^ | 0.166 | 0.166 | 3.63×10^-2^ | 3.63×10^-2^ | 3.63×10^-2^ |
| Illegal trade (products of animal origin) (kg) | 1.49 | 1.49 | 1.49 | 2.69×10^-2^ | 2.69×10^-2^ | 2.69×10^-2^ |
| **COMPARE (First infection; annual probability of at least one event)** |  |  |  |  |  |  |
| Live animals | 2.44×10^-2^ | 2.50×10^-2^ | 2.98×10^-2^ | Negligible | Negligible | Negligible |
| Products of animal origin | 5.73×10^-2^ | 7.48×10^-2^ | 0.121 | 2.23×10^-2^ | 2.24×10^-2^ | 2.26×10^-2^ |
| Wild boar | Negligible | 2.00×10^-5^ | 2.00×10^-5^ | Negligible | Negligible | Negligible |

**Table 1.** Continued.

|  | **Results NLD** | | | **Results FIN** | | |
| --- | --- | --- | --- | --- | --- | --- |
|  | **Base** | **HS1** | **HS2** | **Base** | **HS1** | **HS2** |
| **RRAT (First infection; probability-based risk score)** |  |  |  |  |  |  |
| Live animals | 9.07×10^-3^ | 9.10×10^-3^ | 1.11×10^-2^ | 7.45×10^-10^ | 7.45×10^-10^ | 7.45×10^-10^ |
| Products of animal origin | 0.223 | 0.223 | 0.266 | 2.19×10^-2^ | 2.20×10^-2^ | 2.45×10^-2^ |
| Germplasm^a^ | NA | NA | NA | NA | NA | NA |
| **MINTRISK (Establishment; annual rate)** |  |  |  |  |  |  |
| Live animals | 4.38×10^-4^ | 1.28×10^-3^ | 3.44×10^-3^ | 9.19×10^-8^ | 9.19×10^-8^ | 9.19×10^-8^ |
| Wild boar | 8.91×10^-4^ | 2.82×10^-2^ | 2.82×10^-2^ | 2.82×10^-3^ | 2.82×10^-3^ | 2.82×10^-3^ |
| **IDM (Exposure; risk score)** |  |  |  |  |  |  |
| Live animals | 7.5 | 13 | 18 | 2.5 | 5.5 | 8.5 |
| Products of animal origin^b^ | 12 | 12 | 12 | 12 | 12 | 12 |
| Wild boar | 0.5 | 2.8 | 2.8 | 3.9 | 3.9 | 3.9 |
| Other pathways including transport | 7 | 7 | 7 | 5 | 5 | 5 |

**Table 1.** Continued.

|  | **Results NLD** | | | **Results FIN** | | |
| --- | --- | --- | --- | --- | --- | --- |
|  | **Base** | **HS1** | **HS2** | **Base** | **HS1** | **HS2** |
| **NORA (First infection; risk score)** |  |  |  |  |  |  |
| Live animals | 1.50×10^-3^ | 4.54×10^-2^ | 4.84×10^-2^ | 0 | 0 | 0 |
| Products of animal origin^b^ | 1.52×10^-2^ | 2.02×10^-2^ | 2.02×10^-2^ | 1.05×10^-3^ | 1.50×10^-3^ | 1.50×10^-3^ |
| Wild boar | 1.50×10^-5^ | 1.50×10^-5^ | 1.50×10^-5^ | 1.50×10^-5^ | 1.50×10^-5^ | 1.50×10^-5^ |
| Germplasm^a^ | 0 | 0 | 0 | 0 | 0 | 0 |
| Transport (animal transport vehicles) | 1.00×10^-4^ | 1.00×10^-4^ | 1.00×10^-3^ | 1.00×10^-3^ | 1.00×10^-3^ | 1.00×10^-3^ |
| Goods and traffic other than animals or animal products | 5.00×10^-5^ | 5.00×10^-5^ | 5.00×10^-4^ | 5.00×10^-5^ | 5.00×10^-5^ | 5.00×10^-5^ |
| Feed and bedding | 5.00×10^-4^ | 5.00×10^-4^ | 2.50×10^-3^ | 5.00×10^-4^ | 5.00×10^-4^ | 5.00×10^-4^ |
| Human travel^b^ | 2.64×10^-3^ | 2.64×10^-3^ | 2.64×10^-3^ | 5.00×10^-3^ | 5.00×10^-3^ | 5.00×10^-3^ |
| **SVARRA (Exposure; qualitative probability level)** |  |  |  |  |  |  |
| Live animals | Very low | Very low | Very low | Negligible | Negligible | Negligible |
| Products of animal origin^b^ | Very low | Very low | Very low | Very low | Very low | Very low |
| Wild boar | Negligible | Very low | Very low | Neg-very low | Neg-very low | Neg-very low |
| Germplasm | Negligible | Negligible | Negligible | Negligible | Negligible | Negligible |
| Indirect (transport, human travel, feed and bedding) | Very low | Very low | Very low | Very low | Very low | Very low |

^a^ Volume of trade in pig semen could not reliably estimated from Eurostat (2019).

^b^ Including illegal trade of animal products.

# References

Eurostat, 2019. Comext bulk download. Available from: <https://ec.europa.eu/eurostat/estat-navtree-portlet-prod/BulkDownloadListing?sort=1&dir=comext>. Last accessed 19 April 2019.
