# Supplementary material for: Cross-Validation of Generic Risk Assessment Tools for Animal Disease Incursion Based on a Case Study for African Swine Fever
Source: Front Vet Sci. 2020 Feb 18;7:56. doi: 10.3389/fvets.2020.00056 (PMC7039936; doi:10.3389/fvets.2020.00056)
Supplement: Supplementary file 4 [file Data_Sheet_1.docx]

Supplementary Material 4: Comparison of results of seven generic risk assessment tools with two bespoke models

**
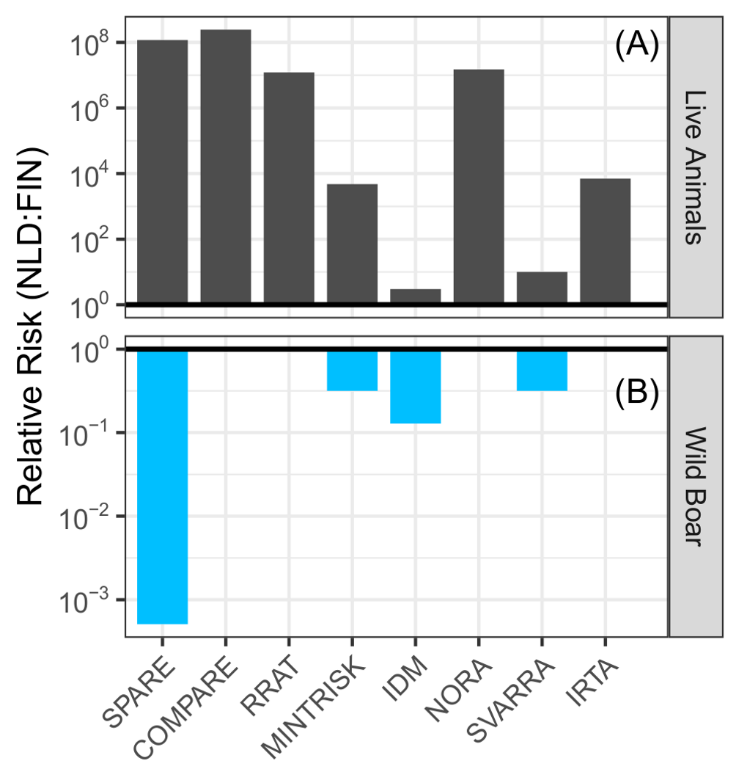
**

**Supplementary Figure 1.** Relative risk of introducing African swine fever into the Netherlands compared to Finland in the baseline scenario (2017 situation) by (a) trade in live animals and (b) movement of wild boar for seven generic risk assessment tools (SPARE, COMPARE, RRAT, MINTRISK, IDM, NORA, SVARRA) and two bespoke models (IRTA_Live Animals and IRTA_Wild Boar). A relative risk above 1 (red line) denotes the Netherlands has a higher risk than Finland, while a relative risk below 1 denotes Finland has a higher risk. Please note the different scales used on the y-axes.

**
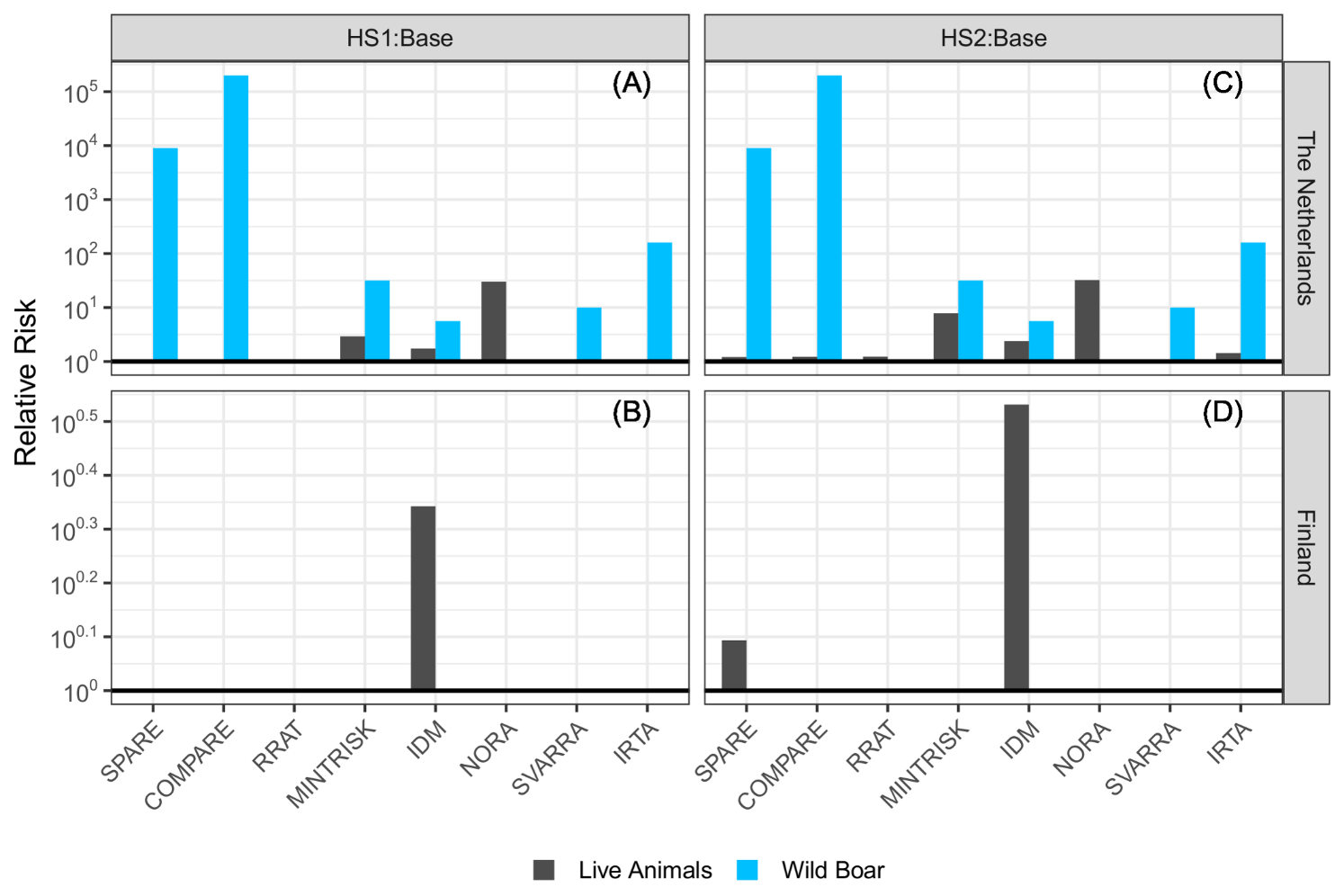
**

**Supplementary Figure 2.** Relative risk of introducing ASF into (a) the Netherlands and (b) Finland in the hypothetical scenario with ASF reported in wild boar in Germany (HS1) and (c) the Netherlands and (d) Finland with ASF reported in wild boar and domestic pigs in Germany (HS2) compared to the baseline scenario (2017 situation) for seven generic risk assessment tools (SPARE, COMPARE, RRAT, MINTRISK, IDM, NORA, SVARRA) and two bespoke models (IRTA_Live Animals and IRTA_Wild Boar). A relative risk of 1 (red line) denotes no increased risk. Please note the different scales used on the y-axes.
