# Supplementary material for: Cross-Validation of Generic Risk Assessment Tools for Animal Disease Incursion Based on a Case Study for African Swine Fever
Source: Front Vet Sci. 2020 Feb 18;7:56. doi: 10.3389/fvets.2020.00056 (PMC7039936; doi:10.3389/fvets.2020.00056)
Supplement: Supplementary file 5 [file Data_Sheet_2.docx]

Supplementary Material 5: Decision criteria to select a generic risk assessment tool

Table 1. Overview of the seven generic risk assessment tools based on criteria to decide which tool suits the need of the risk assessor.

|  | **SPARE** | **COMPARE** | **RRAT** | **MINTRISK** | **IDM** | **NORA** | **SVARRA** |
| --- | --- | --- | --- | --- | --- | --- | --- |
| ***Rationale for the risk assessment*** |  |  |  |  |  |  |  |
| Response to a disease event |  |  |  |  |  | X | X |
| Continuous assessment of incursion risk over a particular time period | X | X | X | X | X |  |  |
| Horizon scanning | X | X | X |  | X |  |  |
| ***Need for prioritization*** |  |  |  |  |  |  |  |
| Pathways |  | X | X | X^a^ |  | X | X |
| Diseases |  |  | X | X | X |  |  |
| Target areas | X | X |  | X |  |  |  |
| ***Endpoint of risk assessment*** |  |  |  |  |  |  |  |
| Entry | X | X | X | X | X | X | X |
| Exposure |  |  |  |  | X |  | X |
| First infection |  | X | X |  |  | X |  |
| Establishment |  |  |  | X |  |  |  |
| Epidemiological consequences |  | X |  | X | X |  |  |
| Economic consequences |  |  |  | X |  | X |  |

Table 1. Continued.

|  | **SPARE** | **COMPARE** | **RRAT** | **MINTRISK** | **IDM** | **NORA** | **SVARRA** |
| --- | --- | --- | --- | --- | --- | --- | --- |
| ***Risk assessment approach*** |  |  |  |  |  |  |  |
| Qualitative |  |  |  |  |  |  | X |
| Semi-quantitative |  |  | X | X | X | X |  |
| Quantitative | X | X |  |  |  |  |  |
| ***Expertise available*** |  |  |  |  |  |  |  |
| Disease expertise only |  |  |  | X | X | X | X |
| Computing expertise only | X |  | X |  |  |  |  |
| Both disease and computing expertise | X | X | X | X | X | X | X |
| ***Time period to perform risk assessment*** |  |  |  |  |  |  |  |
| < 3 days |  |  |  |  | X |  | X |
| < 1 week |  |  | X | X | X |  | X |
| > 1 week | X | X | X | X | X | X | X |

^a^ MINTRISK can compare results across pathways up till the step of establishment, but was not designed to compare across pathways.
